# Machine Learning Attacks‐Resistant Security by Mixed‐Assembled Layers‐Inserted Graphene Physically Unclonable Function

**DOI:** 10.1002/advs.202302604

**Published:** 2023-08-16

**Authors:** Subin Lee, Byung Chul Jang, Minseo Kim, Si Heon Lim, Eunbee Ko, Hyun Ho Kim, Hocheon Yoo

**Affiliations:** ^1^ Department of Electronic Engineering Gachon University 1342 Seongnam‐daero Seongnam 13120 Republic of Korea; ^2^ School of Electronics Engineering Kyungpook National University 80 Daehakro, Bukgu Daegu 41566 Republic of Korea; ^3^ School of Electronics and Electrical Engineering Kyungpook National University 80 Daehakro, Bukgu Daegu 41566 Republic of Korea; ^4^ Department of Energy Engineering Convergence & School of Materials Science and Engineering Kumoh National Institute of Technology 61 Daehakro Gumi‐si Gumi39177 Republic of Korea

**Keywords:** graphene, machine learning attack, physical unclonable function, raman spectroscopy, self‐assembled monolayer

## Abstract

Mixed layers of octadecyltrichlorosilane (ODTS) and 1H,1H,2H,2H‐perfluorooctyltriethoxysilane (FOTS) on an active layer of graphene are used to induce a disordered doping state and form a robust defense system against machine‐learning attacks (ML attacks). The resulting security key is formed from a 12 × 12 array of currents produced at a low voltage of 100 mV. The uniformity and inter‐Hamming distance (HD) of the security key are 50.0 ± 12.3% and 45.5 ± 16.7%, respectively, indicating higher security performance than other graphene‐based security keys. Raman spectroscopy confirmed the uniqueness of the 10,000 points, with the degree of shift of the G peak distinguishing the number of carriers. The resulting defense system has a 10.33% ML attack accuracy, while a FOTS‐inserted graphene device is easily predictable with a 44.81% ML attack accuracy.

## Graphical abstract

Using graphene, this study presents efficient extraction of a security key at a low voltage of 100 mV. By inserting self‐assembled monolayers with different functional groups into the graphene device, an irregular distribution of dipoles in the channel is created, resulting in unique characteristics with sufficient randomness, achieving 50% uniformity, a 45.5% inter‐Hamming distance, and a defense rate of 10.33% against machine learning attacks.

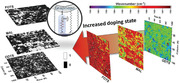

## 1 Introduction

In 1984, Gustavus Simmons suggested using the variability of physical properties to produce an authentication application. This idea led to the development of a physically unclonable function (PUF) that generates a distinct cryptographic key for every device. This system has attracted considerable interest in the security industry.^[^ ^1^ ^]^ A PUF is a hardware component that generates a unique identifier for a specific input and condition and cannot be duplicated.^[^ ^2^ ^]^ This is achieved using the variations in physical properties that form during semiconductor manufacture, which produce a “fingerprint” unique to each device. PUFs are commonly used in high‐security applications, such as encryption, because of their ability to produce a randomly generated output specific to the physical structure of a device.^[^ ^3^ ^]^ The majority of PUFs currently used commercially are developed using silicon integrated circuits (ICs), where both the measurement circuit and the corresponding PUFs are produced through a complementary metal‐oxide‐semiconductor (CMOS) fabrication process based on silicon.^[^ ^4^ ^]^

While PUFs based on silicon ICs have been successful, their manufacturing processes are complex and expensive, requiring the same process steps, including unnecessary ones for PUFs that use physical property variations. As a result, PUFs made with emerging non‐silicon materials are gaining traction as a more viable alternative. PUFs can be fabricated using simplified process steps by inducing randomness in the physical properties. Graphene is a 2D material with unique electronic properties, making it attractive for electronic devices. Graphene‐based electronic devices have been developed for various applications, including sensors,^[^ ^5^ ^]^ transistors,^[^ ^6^ ^]^ and batteries.^[^ ^7^ ^]^ One of the advantages of graphene‐based electronic devices is their low processing cost compared to conventional silicon‐based devices. Graphene‐based devices offer several advantages over conventional silicon‐based devices with respect to simplicity reducing the overall manufacturing time and lowering the production cost. Complex manufacturing processes are needed in conventional silicon‐based devices, including lithography, etching, and doping. These processes involve multiple steps and require expensive equipment, making the production cost of silicon‐based devices high. In contrast, graphene‐based devices can be fabricated using a simple and cost‐effective process.^[^ ^8^ ^]^ Graphene can be grown on various substrates by chemical vapor deposition (CVD)^[^ ^9^ ^]^ or other methods,^[^ ^10^ ^]^ and the graphene layer can be patterned using conventional photolithography techniques. The process of fabricating graphene‐based electronic devices is more straightforward and less expensive than the process of fabricating silicon‐based devices. Moreover, graphene‐based electronic devices have other advantages over silicon‐based devices. Graphene has excellent electrical conductivity, high carrier mobility, and good thermal conductivity, making it suitable for high‐speed and high‐frequency electronic devices.^[^ ^11^ ^]^ More importantly, the interfacial dipole effect in graphene is a promising avenue for exploring new applications in electronics. The interfacial dipole effect is a phenomenon that occurs when graphene comes into contact with a substrate or another material with a different electronegativity than graphene.^[^ ^12^ ^]^ This effect produces a dipole moment at the interface, resulting in a potential difference between the two materials, leading to various phenomena, such as charge transfer, band bending, and doping.^[^ ^13^ ^]^ For example, when graphene is placed on a substrate with a higher electronegativity, such as SiO_2_, the electron density in the graphene layer is shifted towards the substrate, forming a negative charge on the substrate side and a positive charge on the graphene side. This potential difference can modify the electronic properties of graphene, including its carrier concentration and mobility.

Several studies have proposed that emerging PUFs incorporate variations in current and other physical signals as a means of differentiation.^[^ ^14^, ^15^, ^16^ ^]^ In our work, we amplified device deviation by introducing additional doping using materials that possess an opposite dipole to that of graphene. This study introduces an approach for producing secure electronic devices using a mixed‐assembled layer (MAL) inserted into graphene PUFs. Graphene was used to extract a security key efficiently at a low voltage, as low as 100 mV. To implement PUFs with high entropy, it is necessary to have significant device‐to‐device variation. To achieve this, several approaches have been attempted, including 1) variation in filament formation in memristors,^[^ ^17^ ^]^ 2) inherent variation in thin film crystal structures,^[^ ^18^ ^]^ and 3) randomness induced by impurity variation.^[^ ^14^, ^19^ ^]^ Another approach is to utilize an atomically‐thin graphene, which exhibits a strong interfacial dipole effect. This effect occurs when graphene contacts a material with distinct electronegativity compared to the graphene itself. By manipulating the interfaces with materials possessing contrasting polarities, significant variations in electrical characteristics can be achieved. Graphene's unique electrical conductivity in comparison to other materials enables the realization of substantial differences in electrical properties upon contact with substrates with distinct electronegativities. To implement graphene‐based PUFs with high entropy, we used a mixed‐assembled layer of ODTS and FOTS inserted into graphene PUFs. Both self‐assembled monolayers (SAMs) of ODTS and FOTS share the same silane group as the head group, but they differ in their tails: ODTS possesses an alkyl chain, whereas FOTS features a fluoroalkyl chain. These SAMs' tails exhibit non‐identical dipole moments; ODTS has a dipole moment of +0.84 D, while FOTS carries a dipole moment of −3.49 D.^[^ ^20^ ^]^ The significant shift in the Dirac point, which enables the realization of high‐performance PUFs, depends on whether graphene is predominantly bound to ODTS or, conversely, predominantly bound to FOTS. They adjusted the Fermi level by inserting SAMs with different functional groups into the graphene device. This produced an irregular distribution confirmed by measuring the doping state through the electrical characteristics. To assess the security key generated by the device, we employed several indicators, including uniformity, inter‐Hamming distance (inter‐HD), and entropy. These metrics allow us to quantitatively evaluate the randomness and uniqueness of the security key. Each device had unique electrical characteristics because of the irregular distribution of dipoles in the channel. We produced 200 irregular devices with sufficient randomness, 50% uniformity, and a 45.5% inter‐HD, which provided non‐reproducible uniqueness. They measured the peak shift in the G band of a 100 × 100 array of graphene with irregular SAMs using non‐destructive Raman spectroscopy. The security key generated from the data extracted from 10 000 points measured by Raman spectroscopy revealed 50.02% uniformity, a 46.15% inter‐HD, and a defense rate of 10.33% against machine learning (ML) attacks.

## 2 Results and Discussion

**Figure 1:**
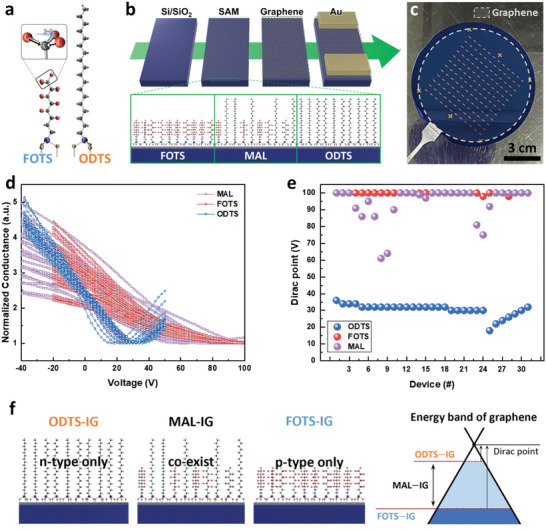
a) Molecular structure of ODTS and FOTS. b) Fabrication process of the PUF device based on graphene on MAL. c) Photograph of a 4‐inch wafer‐scale sample. d) Normalized conductance curve of graphene according to the constituent type of SAM. e) Distribution of Dirac points for each SAM material. f) Schematic diagram of SAM materials and the doping effect of graphene by SAM.

**Figure** **1**a presents the structures of ODTS and FOTS. Both SAMs have the same silane group as the head group but different tails. ODTS has an alkyl chain, while FOTS has a fluoroalkyl chain. The tails of the SAMs have non‐identical dipole moments; ODTS has a dipole moment of +0.84 D, while FOTS has a dipole moment of −3.49 D.^[^ ^20^ ^]^ The dipole moment of SAMs can act as a chemical dopant.^[^ ^20^, ^21^ ^]^ This study examined the doping effect of mixing ODTS and FOTS at the same concentration. The channel material was graphene, a 2D material capable of chemical doping. A graphene transistor was fabricated to extract the Dirac point and analyze the doping effects. Figure 1b presents the process of fabricating the device. We used a 4‐inch Si/SiO_2_ wafer that bonds well with silane groups because the head groups of the SAM materials used are all silane groups, as shown in Figure 1c.^[^ ^22^ ^]^ Graphene was wet‐transferred onto the SAM, and Au was chosen as an electrode because a previous study reported that using Au as the electrode strengthens the p‐type behavior of graphene.^[^ ^23^ ^]^ Au was deposited onto the device by thermal evaporation.^[^ ^24^ ^]^ A bottom‐gate top‐contact graphene transistor was fabricated using a series of processes, as shown in Figure 1b; Figure S1 (Supporting Information). Since graphene transferred onto substrates with different surface energies may exhibit varying doping concentrations and mechanical strains, we examined graphene samples transferred onto ODTS‐, FOTS‐, and MAL‐treated SiO_2_/Si substrates. To separate the effects of mechanical strain and charge doping in graphene, we employed the method previously reported by Lee et al.^[^ ^25^ ^]^ The distributions of doping concentration and mechanical strain are summarized in Table S1 (Supporting Information). The Dirac point is a crucial parameter of graphene because it represents the Fermi level.^[^ ^26^ ^]^ The Dirac points of ODTS‐, FOTS‐, and MAL‐inserted graphene (IG) were compared to analyze the doping effect. The transfer curve was measured, as shown in Figure 1d. The conductance curves of ODTS‐IG and FOTS‐IG were different, while MAL‐IG had relatively diverse conductance curves. The Dirac point was extracted from each transfer curve, as shown in Figure 1e. The Dirac point of FOTS‐IG was higher than 100 V, while that of ODTS‐IG was equal to 30.9 ± 3.8 V, respectively. In contrast, the Dirac point of MAL‐IG had a minimum of 61 V and a maximum of 100 V. The variation in the Dirac points observed in ODTS‐, FOTS‐, and MAL‐IG can be explained by the type and distribution of SAMs, as shown in Figure 1f. ODTS‐IG had only an n‐type dopant, while FOTS‐IG had only a p‐type dopant. In addition, to scrutinize the specific Fermi level of each SAM‐inserted graphene, ultraviolet photoelectron spectroscopy (UPS) analysis was performed, as shown in Figure S2 (Supporting Information). Based on the values of each cut‐off energy, it was observed that the work functions of ODTS‐IG, MAL‐IG, and FOTS‐IG were calculated to be 4.5 eV, 4.9 eV, and 5.1 eV, respectively, which aligns with the trend depicted in Figure 1d,e. ODTS‐IG exhibits p‐type behavior because of the residual poly(methyl methacrylate) (PMMA) remaining from the graphene transfer process. PMMA acts as a p‐type dopant and produces stronger p‐doping than the ODTS treatment, resulting in a positive Dirac voltage. As SAMs are inserted into graphene, the Fermi level strongly becomes p‐type in FOTS‐IG compared to the Fermi level in ODTS‐IG. In MAL‐IG, p‐ and n‐type dopants coexist, resulting in a Fermi level between ODTS‐ and FOTS‐IG.

Each transistor in the MAL‐IG device exhibits a distinct Dirac point, suggesting that the doping states and levels vary because of the random distribution of ODTS and FOTS. This randomness is an inherent characteristic of the physical entity and a result of the semiconductor manufacturing process during spin coating. This feature suggests that MAL‐IG can be used as a PUF device, and its security properties can be assessed. The devices were arranged in a 12 × 12 grid, and the output of each device was converted into a binary digital value to produce a hardware‐based security system. The current value of each device was used as the reference for each binary bit. The current data was divided into 0 and 1 based on the median current value to produce the dichotomous data for each bit.

**Figure 2:**
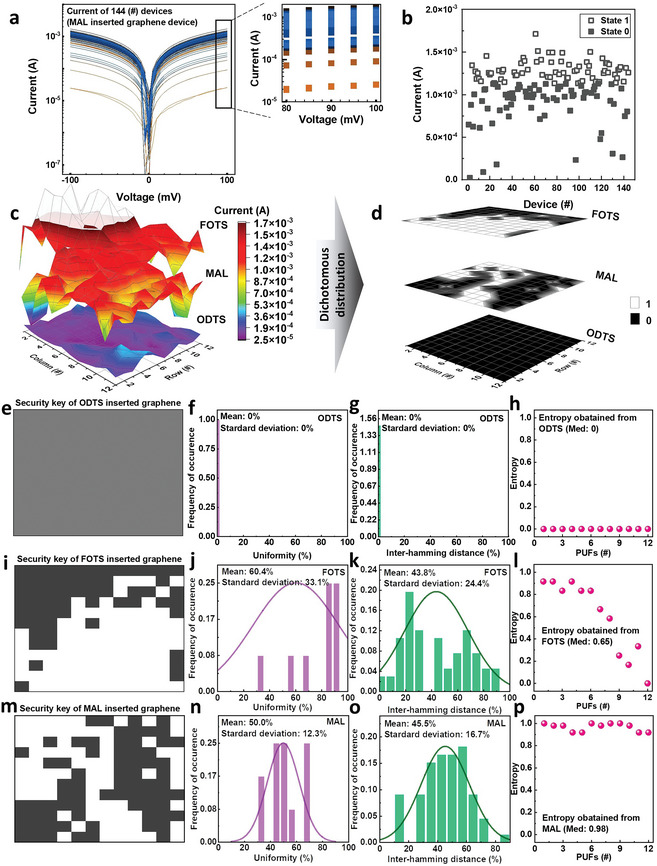
a) Current from 144 devices when a voltage of −100 mV to 100 mV applied to the graphene on the MAL. Magnified graph of currents from 80 mV to 100 mV are included. b) Current and state distribution at 100 mV in 144 devices. c) Mapping the current generated at 100 mV for each material under graphene to a 12 × 12 array. d) Result of mapping the current to 12 × 12 arrays by dividing it into 1 and 0. e) Current‐based security key generated from graphene on ODTS layer and its f) uniformity, g) inter‐HD, and h) entropy, respectively. i) Current‐based security key generated from the graphene on FOTS layer and its j) uniformity, k) inter‐HD, and l) entropy, respectively. m) Current‐based security key generated from the graphene on the MAL layer and its n) uniformity, o) inter‐HD, and p) entropy, respectively.

The median value of the current was determined to establish a standard state. The currents were obtained by sweeping the current–voltage characteristics between two Au electrodes from −100 mV to 100 mV, as shown in **Figure** **2**a. All measured curves are given in each Supporting Information Figure S3–S5 (Supporting Information) representing ODTS‐IG, MAL‐IG, and FOTS‐IG, respectively. The current distribution of the array of MAL‐IG was wide, with a folded level of ≈67.6 A current range of 25.3 µA to 1.71 mA was observed at 100 mV. Figure 2b shows the current measured in each device at 100 mV. All currents were divided into dichotomous values of either 0 or 1. The median value of 1,130 µA was determined by considering all the currents obtained.

The security key was obtained by matching the current generated by each device in the 12 × 12 array to its actual position. The current distribution map, shown in Figure 2c, was produced by mapping the current according to the various SAMs, which determine the doping state by their functional groups at each device in the array. The current distribution was higher for FOTS‐IG than ODTS‐IG because of the negative dipole‐based FOTS and ODTS layers inserted in graphene. MAL‐IG had a disordered current distribution because of the co‐existence of both SAMs. The MAL‐IG array graph was mapped, as shown in Figure S6 (Supporting Information). Figure 2d presents the dichotomous states of the generated current divided into 0 and 1. ODTS‐IG, which had a low current, was assigned a value of 0 compared to the median value of the MAL‐IG device. FOTS‐IG had a security key in which state 1 was concentrated in a specific area, while MAL‐IG had a security key in which states 0 and 1 were arranged randomly.

We first evaluated two parameters to assess the security key obtained: uniformity and inter‐HD. The method for calculating these two parameters is as follows:

(1) $$\mathrm{uniformity}\;\;\left(\%\right)=\frac{1}{n}\;\sum_{i\;=\;1}^{n}r_{i}\times 100\;$$

where ***r***_***i***_ is the number of a bit ‘0′, and ***n*** is the string length of the security key.

(2) $$\mathrm{inter}-\mathrm{HD}\;\left(\%\right)\;=\frac{2}{k\left(k-1\right)}\;\sum_{i-1}^{k-1}\sum_{j\;=\;i+1}^{k}\frac{HD\left(r_{i},\;r_{j}\right)}{n}\times 100\%$$

where ***r***_***i***_ and ***r***_***j***_ indicate the responses of the security key ‘*i*’ and “*j*”, respectively, in response to a given challenge, and *k* and *n* are the number of security keys and the string length of the security key, respectively. Uniformity is a parameter representing the ratio of states 0 to 1. A uniformity of 50% indicates the high randomness of the response bit. The pattern is monotonic and predictable when the states are clustered on 0 or 1. That is, a uniformity of 50% is ideal as a security device. Inter‐HD represents the difference in pattern between the units when the security key is divided into units and analyzed. For example, the inter‐HD is 0% if the pattern arrangements of the two units match and is 100% if they are opposite. The patterns were normalized and predicted when inter‐HD approaches 0% or 100%. Hence, a 50% inter‐HD is ideal as a security device. The security key based on ODTS‐IG was evaluated. This key could not be used as a security device because it consisted only of state 0 as shown in Figure 2e which shows 0% uniformity (Figure 2f), inter‐HD (Figure 2g) and entropy (Figure 2h). The FOTS‐IG‐based security key was also evaluated, but it could not be used as a hardware‐based security key because of the low uniformity and high inter‐HD. The FOTS‐IG‐based security key consisted of states 0 and 1, with state 1 being partially biased.

The extracted uniformity of the FOTS‐IG‐based security key was calculated to be 60.4 ± 33.1%, indicating more state 1, with many units having a uniformity of 80% or more. The inter‐HD of the FOTS‐IG‐based security key was calculated to be 43.8 ± 24.4%, indicating a large deviation, with large statistical values of ≈25% and 65% in the actual distribution. We also investigated the entropy value, which was equal to 65% (Figure 2l). Based on these results, the FOTS‐IG‐based security key was found to be unsuitable as a hardware‐based security key. Sequentially, the MAL‐IG‐based PUFs were investigated. Figure 2m shows the MAL‐IG‐based security keys. The distribution of 0 and 1 states was even and random compared to the two previous security keys inserted with only a single SAM. The uniformity of each unit was calculated and collected, and Figure 2n shows the results. The uniformity was 50.0 ± 12.3%, indicating a relatively small deviation from the ideal average value, and the distribution was concentrated in the 30% to 70% range, suggesting that all units had a uniformity close to 50%. Figure 2o shows the result of calculating and collecting the inter‐HD between each unit. An inter‐HD of 45.5 ± 16.7% was observed, with average values close to 50% and relatively small deviations compared to the devices inserted with a single SAM. The actual distribution also showed a distribution centered on 50%, suggesting that each inter‐HD was difficult to replicate. In addition, 98% entropy was obtained from the MAL‐inserted graphene which shows the highest value rather than a single SAM‐inserted graphene device in Figure 2p. High entropy indicates a greater level of unpredictability and randomness. The results indicated that the MAL‐IG‐based security key has ideal uniformity and an inter‐HD compared to the two sole SAM‐inserted security keys observed previously. Hence, it is expected to be used as a PUF security device. In addition, we extended the fabrication of PUF security keys in the same process by inserting two SAMs beneath the graphene layer. Figure S7 (Supporting Information) exhibits the resulting increased pixels in a 20 × 20 array. Each security key displayed distinct characteristics, resulting in a uniformity of 50% (Figure S7b, Supporting Information), an inter‐HD of 49.3% (Figure S7c, Supporting Information), and an entropy of 93% (Figure S7d, Supporting Information), respectively. Further, to assess the long‐term stability of the PUF, we performed a re‐measurement of the identical device after a duration of 293 days. Our observations indicate that the security key exhibits a 100% match when compared to the original MAL‐inserted graphene device, as shown in Figure S8 (Supporting Information). We also investigated the stability of the graphene devices as shown in Figure S9 (Supporting Information). The ODTS‐IG and FOTS‐IG were prepared and their electrical properties were used to assess stability. First, the resistance of both materials remained constant for 10^5^ seconds, indicating strong ambient stability. Second, even at a high temperature of 100°C, both ODTS‐IG and FOTS‐IG showed no significant degradation, demonstrating their stability under elevated thermal conditions. Lastly, moisture exposure had minimal impact on the electrical properties of ODTS‐IG and FOTS‐IG, as evidenced by similar *I–V* curves in both ambient air and high humidity (80% relative humidity). The stability of graphene on ODTS can be attributed to the hydrophobic surface (Figure S10, Supporting Information).^[^ ^27^ ^]^

**Figure 3:**
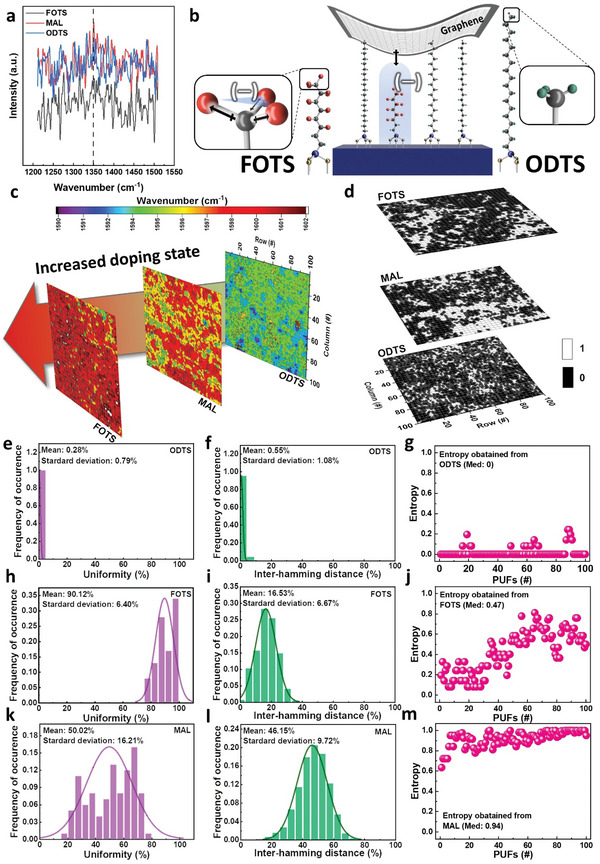
a) D band region at each SAM‐inserted graphene. b) Schematic diagram of MAL‐IG and their dipole states. c) Mapping shifted value in the G band depending on the doping concentration in each array and d) their dichotomously converted value. Analysis results for e) uniformity, f) inter‐HD, and g) entropy of ODTS‐IGD PUF, h) uniformity, i) inter‐HD, and j) entropy of FOTS‐IGD PUF, and k) uniformity, l) inter‐HD, and m) entropy of MAL‐IGD PUF.

Based on graphene, which is atomically thin,^[^ ^28^ ^]^ the type of molecule can be verified by Raman spectroscopy, which absorbs as much energy as the difference in the energy level of electrons of the molecule when a specific molecule is irradiated with a laser. These methods can provide information on the molecules from the nanometer‐scale material. The resonance by incident light has an extensive range of energies because graphene has an energy bandgap shape with the pointed parts of two cones facing each other. As a result, graphene has a high‐intensity Raman signal despite having an atomic‐layer thickness.^[^ ^29^ ^]^ As mentioned above, the Fermi level of graphene was modulated by inserting dipole‐inducing SAMs. Although graphene was doped, the doping effect should not be the defect site, and the performance should not be suppressed. Therefore, the *D* band, which is the defect band originating from the vibrational process caused by the resonance of the phonon lattice at 1350 cm^−1^, was not observed,^[^ ^30^ ^]^ as shown in **Figure** **3**a, and no additional defects were observed in the D/G ratio (Figure S11, Supporting Information) and atomic force microscopy (AFM) (Figure S12, Supporting Information). Successful doping of graphene without defects was achieved based on the abovementioned point of view. In addition, various properties of graphene, such as the number of layers,^[^ ^31^ ^]^ chemical structure,^[^ ^32^ ^]^ strain,^[^ ^33^ ^]^ and doping state,^[^ ^34^ ^]^ can be known by the band generated in Raman spectroscopy. The characteristics of the graphene device exhibited significant changes attributed to the dipole effect caused by the molecules of the functional group of SAMs, as well as the G band shift resulting from the alteration in the number of carriers. Among the properties, the peak at the G band, which is the primary mode of graphene, indicates the in‐plane E_2g_ phonon peak at the Brillouin zone.^[^ ^35^ ^]^ Energy conversion is known by the degree of shift of the G band peak.

The doping state was observed by shifted energy conversion, which originated from intentional modulation of the Fermi level by doping SAM at graphene. As shown in Figure 3b, an ODTS‐inserted graphene device (IGD) behaves similarly to intrinsic graphene because of the deficient doping effect of the carbon chain. In contrast, FOTS contributes to p‐doping with the strong dipole moment of the CF_3_ functional groups.^[^ ^36^ ^]^ ODTS is composed of an alkyl chain, which induces intrinsic doping, but FOTS causes additional carrier doping because of fluorine as a functional group. Doping through electron–phonon coupling causes a change in frequency at the graphene lattice. ^[^ ^37^ ^]^ As mentioned above, the peak at the G band was shifted by the change in energy. Therefore, the G band is blue‐shifted according to the doped carrier density.^[^ ^38^ ^]^

In Figure 3c, a shifted peak was investigated at 100 × 100 points. The peaks shifted according to the doping state: single ODTS‐IGD showed a wavenumber close to the intrinsic graphene peak, but an increased wavenumber was observed in FOTS‐IGD because of the doping effect on graphene. The MAL‐IGD showed a shorter shift than FOTS‐IGD but more than ODTS‐IGD because of the median concentration of MAL. Figure S13 and S14 (Supporting Information) show the G band from Raman spectroscopy measured for each device and the normal distribution graph of the corresponding data, respectively. The Raman spectroscopy wavenumbers were converted to digital values at the MAL‐IGD by assigning the states based on a comparison with the median value of 10000 samples. State 1 was assigned if the value was higher than the median, and state 0 was assigned if it was lower than the median. This resulted in a dichotomously divided discrete value implemented in an array, as shown in Figure 3d.

The difficulty of making predictions was determined by adopting uniformity and inter‐HD as performance indices. At the ODTS‐IGD device, uniformity of 0.28% (Figure 3e) was observed, with state 0 distributed widely. The uniformity was evaluated at 1.08% of the inter‐HD (Figure 3f), indicating high predictability with low uniqueness. In contrast, the FOTS‐IGD device showed consistent performance with a high uniformity of 90.12% (Figure 3h), divided primarily dichotomously on state 1. The inter‐HD value was also evaluated at 16.53% (Figure 3i). On the other hand, the MAL‐IGD, with two SAM materials of different dipoles coexisting, had a uniformity of 50.02% with high randomness as shown in Figure 3k, almost equally distributed between 0 and 1. The inter‐HD value of 46.15% is shown in Figure 3l and this value is close to 50%, and had a non‐identical value, making it difficult to predict when comparing dichotomously divided values. Furthermore, the entropy values obtained from the ODTS‐IGD, FOTS‐IGD, and MAL‐IGD PUFs were 0%, 47%, and 94%, respectively, as shown in Figure 3g, 3j, and 3m. The variation within the device was examined using two methods: utilizing the electrical characteristics of the device and the physical dipole characteristics. This was necessary because of the irregular distribution of SAM present on a single die.

**Figure 4:**
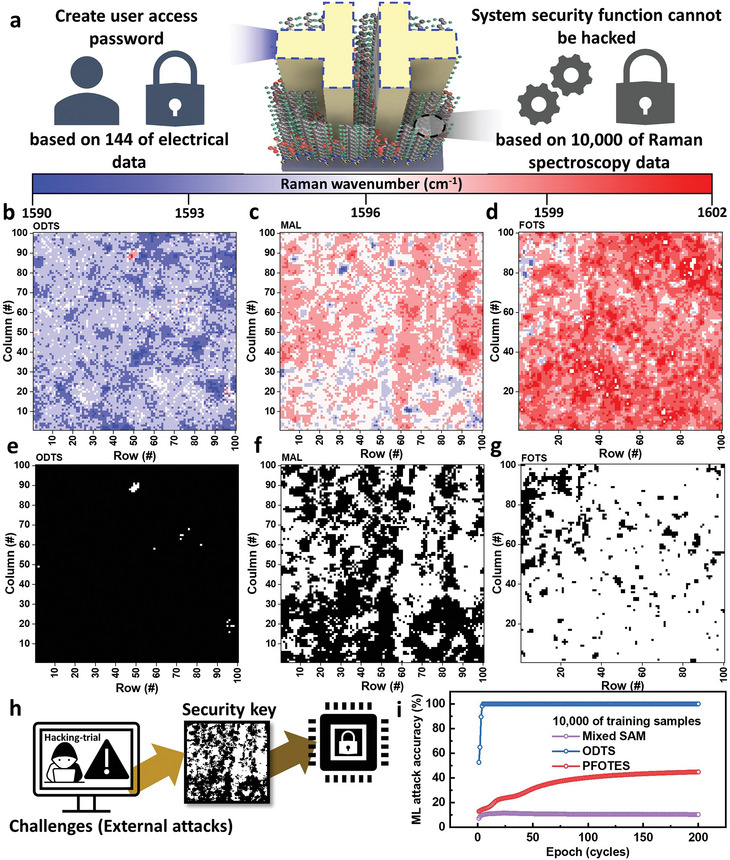
a) Schematic diagram of the suggested dual security system for a user access password and systematic security. G peak mapping (100 µm × 100 µm) measured by Raman spectroscopy at b) ODTS, c) MAL, and d) FOTS‐IGD PUF and their digitally converted value to e) ODTS‐, f) MAL‐ and g) FOTS‐IGD PUF. h) Schematic diagram of a systematic defense system for external attacks and i) machine learning accuracy at each SAM‐IGD PUF.

**Figure** **4**a proposes a dual security mode using 144 electrical characteristics in a single device to produce a security key that only individual users can access. A defense system was implemented by the unpredictable distribution of Raman spectroscopy based on 10 000 points, which was proven by machine learning (ML) attack tests. The degree of shift was observed by the doping state when fabricating a security key for the wavenumber of the shifted G peak, as shown in Figure 4b–d. A single ODTS‐IGD showed a rare shift with a value close to the G peak of intrinsic graphene, while the FOTS‐IGD showed a higher shift than the ODTS‐inserted state. The degree of shift increased as the FOTS concentration increased. In the digitally converted value of the wavenumber, single ODTS and FOTS‐IGD were involved only in each SAM material. Hence, the values were biased toward a single state. On the other hand, the G peak of MAL‐IGD was distributed over a large range at the wavenumber of the G peak from single ODTS‐IGD to single FOTS‐IGD. The security key was produced. Based on the extracted data, such as the quick response (QR) code shown in Figure 4e–g. Typically, the cross‐bar array generates a strong PUF generation to multiply the database. In this study, however, a strong PUF was produced using the information at only one die. Regarding the performance of the Raman‐based strong PUF, as in the figure‐of‐merits mentioned above, each state value was biased in the single SAM‐inserted graphene device, showing an easily predictable value. A PUF test was conducted using 10 000 of data for the ML attack. Regression models are well known for ML attacks on various strong PUFs^[^ ^14^, ^39^ ^]^ because of their ability to generate PUF models directly using the supervised learning algorithm. The vulnerability of a strong PUF to ML attacks was assessed by implementing a multilayer perceptron (MLP) with 100 input neurons, 200 neurons in the first hidden layer, 200 neurons in the second hidden layer, and 50 output neurons. The MLP was trained using a 100‐bit challenge and 100‐bit response, which were used as features and labels, respectively. A sigmoid activation function was used to enhance the accuracy of the MLP, and the synapse weights were initialized using a normal distribution with a mean of 0 and a standard deviation of the reciprocal square root of the number of nodes. The MLP was trained using 10 000 challenge‐response pairs, and the ML attack test was performed by varying the number of epochs.

The ML attack showed that it was very difficult to model the MAL‐IGD PUF with an attack rate of only 10.33% for the partially randomly doped graphene device. Hence, the MAL‐IGD PUF is highly resistant to ML attacks, as shown in Table S2 (Supporting Information). The ML attack accuracy of 10.33% showed a competitive prediction resistance value compared to those in previously reported PUFs models.^[^ ^40^ ^]^ Furthermore, to compare the performance of our PUF with other graphene‐based PUFs, we examined their respective mechanisms, operational voltages, and machine‐learning prediction accuracies. Notably, our study achieved the lowest prediction ratio of 10.33%, as presented in Table S3 (Supporting Information). Such a security device with very unpredictable high defense rates can be applied to systematic defense against attacks like distributed denial of service (DDoS), and personal password generation. With the data obtained from an electronic device, the MAL‐IGD PUF can generate a non‐replicable and difficult‐to‐predict key, such as a user's password. This system was resistant to external attacks because of the extensive data set and the extreme difficulty in predicting the shift in the G peak of Raman spectroscopy, which was measured at 10 000 points in terms of the irregularly distributed SAM and acted irregularly because of the dipole. Thus, this paper proposes a dual‐mode hardware‐based security system that can provide systemic security and generate a user password in a four‐inch wafer‐scale device.

## 3 Conclusion

This study achieved non‐uniform electrical characteristics by doping graphene with two MALs with different functional groups to modulate the Fermi level. The device was fabricated using a MAL, intentionally inducing an irregular dipole distribution to produce dual‐mode security keys. The doping state was determined by the self‐variation of MAL, and an unpredictable key with 50% uniformity and 45.5% inter‐HD performance was achieved by measuring the electrical characteristics of a 12 × 12 array. Raman spectroscopy was used to measure the singularity of the device at 100 × 100 points, and the G band peak of graphene was used to distinguish the effect of the dipole. Single ODTS‐IGD was similar to intrinsic graphene, and the single FOTS‐IGD had a larger G band shift because of the p‐doping effect of the CF_3_ functional group. MAL‐IGD, in which two SAMs coexist, showed a moderate shift because of the dipole moment of each SAM. The strong PUF exhibited randomness with 50.02% uniformity and 46.15% inter‐HD, with each state distributed unpredictably and non‐uniformly. The ML attack accuracy of the strong PUF resulted in a high defense rate of 10.33%. A hardware‐based high‐security system could be implemented using this approach. However, the readout speed of our Raman‐based PUF is not enough to meet the requirement for registration, thus further work is being conducted to improve the readout speed using the advanced Raman systems.^[^ ^41^ ^]^ As this study proposed a dual‐mode security system using irregular electrical characteristics and Raman spectroscopy to modulate the Fermi level of doped graphene, it is believed that this new approach to dual‐mode security system manufacturing with make a valuable contribution to security systems.

## 4 Experimental Section

### Materials

Octadecyltrichlorosilane (CH_3_(CH_2_)_17_SiC_l3_) (ODTS) (≥90%), 1H,1H,2H,2H‐perfluorodecyltriethoxysilane (C_14_H_19_F_13_O_3_Si) (FOTS) (98%), and chlorobenzene (C_6_H_5_Cl) (≥99.5%) were supplied by Sigma–Aldrich (USA). SiO_2_‐coated Si substrate (4‐inch) was used as the substrate. Au was used as the electrodes.

### Synthesis and Transfer of Graphene

Monolayer graphene was deposited by low‐pressure chemical vapor deposition. Copper foil was heated to 1030 °C under flowing H_2_ at 10 sccm. After reaching the temperature, CH_4_ with a flow rate of 30 sccm was added and maintained for 30 min, followed by rapid cooling. The conventional transfer process was adopted using polymethylmethacrylate (PMMA, Mw = 340 kg mol^−1^) to transfer the CVD‐grown graphene onto SAM‐treated Si substrates, as reported elsewhere.^[^ ^27^ ^]^

### Preparation of Precursor Solution

The self‐assembled monolayer (SAM) materials used in this study were ODTS (≥90%) and FOTS (98%). The SAM materials were dissolved in chlorobenzene (≥99.5%) (Sigma–Aldrich).

### SAM Solution and PUF Fabrication Process

The material was coated on a four‐inch wafer with a 300 nm SiO_2_ layer. The substrates were cleaned sequentially with ethanol and isopropyl alcohol (IPA) in bath sonication and dried using blowing N_2_. The substrate was ozone treated to form an o─bond. The solutions were synthesized by dissolving SAM materials (ODTS, FOTS, and mixing ODTS and FOTS) in chlorobenzene and spin‐coated at 3 000 rpm for 10 s using 6.48 mL of solution on a 4‐inch wafer. The device was annealed on a hotplate at 120 °C for 20 min to align the SAM materials. A 100 nm Au layer was deposited by thermal evaporation.

### Characterization

Raman spectroscopy (UniRAM, UniNanoTech) was conducted to investigate the SAM‐inserted graphene film. The transfer curve was measured using a DC source meter (Keithley 2450), and the device property was measured using a Keithley 4200A device to measure the electrical characteristics of the SAM‐inserted graphene device.

## Conflict of Interest

The authors declare no conflict of interest.

## Supporting information

Supporting Information

## Data Availability

The data that support the findings of this study are available on request from the corresponding author. The data are not publicly available due to privacy or ethical restrictions.

## References

[1] a) G. J. Simmons , presented at Advance in Cryptology: Proceedings of Crypto 83, 1984;

[2] Y. Gao , S. F. Al‐Sarawi , D. Abbott , Nat. Electron. 2020, 3, 81.

[3] a) C. Herder , M.‐D. Yu , F. Koushanfar , S. Devadas , Proc.IEEE 2014, 102, 1126;

[4] a) M. Bhargava , K. Mai , presented at 2014 Design, Automation & Test in Europe Conference & Exhibition (DATE), 2014;

[5] a) H. J. Yoon , J. H. Yang , Z. Zhou , S. S. Yang , M. M.‐C. Cheng , Sens. Actuators, B 2011, 157, 310;

[6] a) F. Urban , G. Lupina , A. Grillo , N. Martucciello , A. Di Bartolomeo , Nano Express 2020, 1, 010001;

[7] a) C. Wang , Y. Zeng , X. Xiao , S. Wu , G. Zhong , K. Xu , Z. Wei , W. Su , X. Lu , J. Energy Chem. 2020, 43, 182;

[8] a) S. Kumar , S. Kaushik , R. Pratap , S. Raghavan , ACS Appl. Mater. Interfaces 2015, 7, 2189; 25597697 10.1021/am5084122

[9] a) G. Deokar , J. Avila , I. Razado‐Colambo , J.‐L. Codron , C. Boyaval , E. Galopin , M.‐C. Asensio , D. Vignaud , Carbon 2015, 89, 82;

[10] a) Y. Si , E. T. Samulski , Chem. Mater. 2008, 20, 6792;

[11] a) M. A. Worsley , P. J. Pauzauskie , T. Y. Olson , J. Biener , J. H. Satcher Jr , T. F. Baumann , J. Am. Chem. Soc. 2010, 132, 14067; 20860374 10.1021/ja1072299

[12] a) N. Nagamura , H. Fukidome , K. Nagashio , K. Horiba , T. Ide , K. Funakubo , K. Tashima , A. Toriumi , M. Suemitsu , K. Horn , Carbon 2019, 152, 680;

[13] a) O. L. Monti , J. Phys. Chem. Lett. 2012, 3, 2342; 26292112 10.1021/jz300850x

[14] a) A. Dodda , S. Subbulakshmi Radhakrishnan , T. F. Schranghamer , D. Buzzell , P. Sengupta , S. Das , Nat. Electron. 2021, 4, 364;

[15] a) J. Wu , X. Liu , X. Liu , Z. Tang , Z. Huang , W. Lin , X. Lin , G. Yi , Chem. Eng. J. 2022, 439, 135601;

[16] N. Sun , Z. Chen , Y. Wang , S. Wang , Y. Xie , Q. Liu , Nat. Commun. 2023, 14, 2185. 37069144 10.1038/s41467-023-37588-5 PMC10110537

[17] a) H. M. Ibrahim , H. Abunahla , B. Mohammad , H. AlKhzaimi , Sci. Rep. 2022, 12, 8633; 35606367 10.1038/s41598-022-11240-6 PMC9126908

[18] a) Y. Lin , H. Zhang , J. Feng , B. Shi , M. Zhang , Y. Han , W. Wen , T. Zhang , Y. Qi , J. Wu , Small 2021, 17, 2100244; 10.1002/smll.202100244 34160145

[19] a) J. Zhang , Z. Guo , S. Zhang , Z. Cao , R. Li , J. Cao , M. Song , M. Wan , J. Hong , L. You , Appl. Phys. Lett. 2020, 116;

[20] a) M. Nakano , I. Osaka , K. Takimiya , Adv. Mater. 2017, 29, 1602893; 10.1002/adma.201602893 28042890

[21] D. H. Kang , M. S. Kim , J. Shim , J. Jeon , H. Y. Park , W. S. Jung , H. Y. Yu , C. H. Pang , S. Lee , J. H. Park , Adv. Funct. Mater. 2015, 25, 4219.

[22] Y. Sun , A. R. Negreira , J. Meersschaut , I. Hoflijk , I. Vaesen , T. Conard , H. Struyf , Z. Tokei , J. Boemmels , M. Moinpour , Microelectron. Eng. 2017, 167, 32.

[23] P.‐F. Wang , Y. Liu , J. Yin , W. Ma , Z. Dong , W. Zhang , J.‐L. Zhu , J.‐L. Sun , J. Mater. Chem. C 2019, 7, 887.

[24] P. Khomyakov , G. Giovannetti , P. Rusu , G. v. Brocks , J. Van den Brink , P. J. Kelly , Phys. Rev. B 2009, 79, 195425.

[25] J. E. Lee , G. Ahn , J. Shim , Y. S. Lee , S. Ryu , Nat. Commun. 2012, 3, 1024. 22929781 10.1038/ncomms2022

[26] X. Wang , Y. Chen , G. Wu , J. Wang , B. Tian , S. Sun , H. Shen , T. Lin , W. Hu , T. Kang , Nanotechnology 2018, 29, 134002. 29339566 10.1088/1361-6528/aaa852

[27] E. Lee , H. Lim , N.‐S. Lee , H. H. Kim , Sens. Actuators, B 2021, 347, 130579.

[28] M. E. Suk , N. R. Aluru , J. Phys. Chem. Lett. 2010, 1, 1590.

[29] P. Avouris , Z. Chen , V. Perebeinos , Nat. Nanotechnol. 2007, 2, 605. 18654384 10.1038/nnano.2007.300

[30] J.‐H. Chen , W. G. Cullen , C. Jang , M. Fuhrer , E. D. Williams , Phys. Rev. Lett. 2009, 102, 236805. 19658959 10.1103/PhysRevLett.102.236805

[31] Y. Hao , Y. Wang , L. Wang , Z. Ni , Z. Wang , R. Wang , C. K. Koo , Z. Shen , J. T. Thong , Small 2010, 6, 195. 19908274 10.1002/smll.200901173

[32] L. Panchakarla , A. Govindaraj , C. Rao , Inorg. Chim. Acta 2010, 363, 4163.

[33] M. Huang , H. Yan , T. F. Heinz , J. Hone , Nano Lett. 2010, 10, 4074. 20735024 10.1021/nl102123c

[34] F. Cerdeira , M. Cardona , Phys. Rev. B 1972, 5, 1440.

[35] F. Tuinstra , J. L. Koenig , J. Chem. Phys. 1970, 53, 1126.

[36] M. Çakır , Coatings 2017, 7, 37.

[37] a) M. Lazzeri , F. Mauri , Phys. Rev. Lett. 2006, 97, 266407; 17280442 10.1103/PhysRevLett.97.266407

[38] B. Tang , H. Guoxin , H. Gao , Appl. Spectrosc. Rev. 2010, 45, 369.

[39] a) U. Rührmair , J. Sölter , F. Sehnke , X. Xu , A. Mahmoud , V. Stoyanova , G. Dror , J. Schmidhuber , W. Burleson , S. Devadas , IEEE tran. on info. foren. and sec. 2013, 8, 1876;

[40] a) S. Park , J. So , Appl. Sci. 2020, 10, 8079;

[41] Y. Gu , C. He , Y. Zhang , L. Lin , B. D. Thackray , J. Ye , Nat. Commun. 2020, 11, 516. 31980613 10.1038/s41467-019-14070-9 PMC6981139

