# Supplementary material for: Machine Learning Attacks‐Resistant Security by Mixed‐Assembled Layers‐Inserted Graphene Physically Unclonable Function
Source: Adv Sci (Weinh). 2023 Aug 16;10(30):2302604. doi: 10.1002/advs.202302604 (PMC10602573; doi:10.1002/advs.202302604)
Supplement: Supplementary file 1 — Supporting Information [file ADVS-10-2302604-s001.pdf]

ADVANCED SCIENCE
Open Access

Supporting Information

for *Adv. Sci.*, DOI 10.1002/advs.202302604

Machine Learning Attacks-Resistant Security by Mixed-Assembled Layers-Inserted Graphene Physically Unclonable Function

Subin Lee, Byung Chul Jang, Minseo Kim, Si Heon Lim, Eunbee Ko, Hyun Ho Kim and Hocheon Yoo**

Supporting information

Machine Learning Attacks-Resistant Security by Mixed-Assembled Layers-Inserted Graphene Physically Unclonable Function

Subin Lee, Byung Chul Jang, Minseo Kim, Si Heon Lim, Eunbee Ko, Hyun Ho Kim, and Hocheon Yoo

Figure S1. Fabrication process of graphene··p. 35

Table S1. Doping and mechanical strain of SAM-inserted graphene············p. 36

Figure S2. UPS measurement at SAM-inserted graphene. ······················· **P. 37**

Figure S3. ODTS-inserted graphene characteristics of 144 devices ········ **p. 38-41**

Figure S4. MAL-inserted graphene characteristics of 144 devices·········· **p. 42-45**

Figure S5. FOTS-inserted graphene characteristics of 144 devices ········ **p. 46-49**

Figure S6. Current mapping of MAL-inserted graphene ························**p. 50**

Figure S7. Security key and evaluation at 20 × 20 array device. ··················**p. 51**

Figure S8. Long-term stability of device. ···**p. 52**

Figure S9. Stability of SAM materials aspect to the temperature and moisture environment··**p. 53**

Figure S10. The gate-voltage-dependent at graphene······························ **p. 54**

Figure S11. Correlation between the frequencies of the G-band (ω_G) and 2D-band (ω_{2D}) of graphene. ·· **P. 55**

Figure S12. AFM image of SAM-inserted graphene.······························ **P. 56**

Figure S13. Raman spectroscopy at G band of graphene by SAM···············**p. 57**

Figure S14. Raman spectroscopy distribution graph of graphene by SAM·····**p. 58**

Table S2. Machine learning attack prediction rate by sorts of inserted SAM··**p. 59**

Table S3. Mechanism and evaluation of various graphene PUF **P. 60**

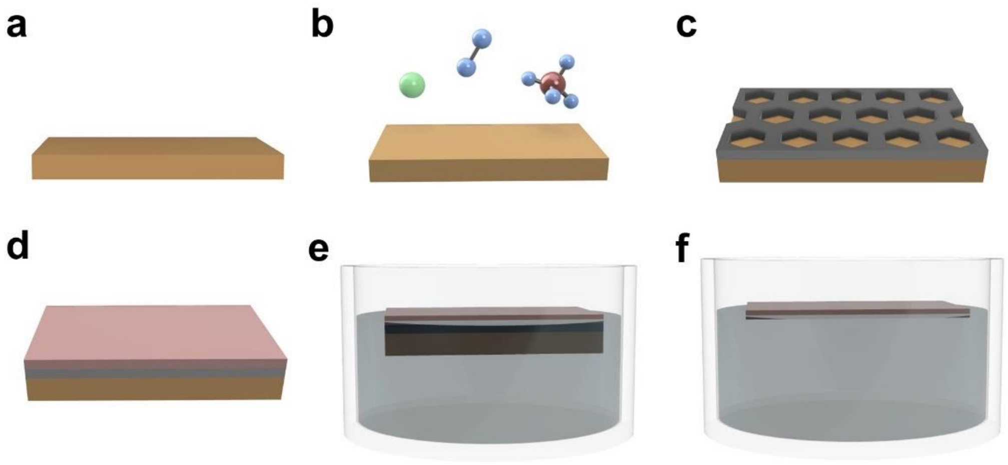

Figure S1. Schematic diagram of graphene synthesis; a) cleaning substrate, b) growing graphene by CVD. c) Schematic diagram of synthesized graphene. d) Coating PMMA on graphene and e) erasing PMMA to leave graphene. f) Wet-transfer graphene.

Table S1. Doping and mechanical strain of CVD graphene transferred onto three different surfaces.

Device	Doping concentration ($\times 10^{12}$ cm^{-2})	Strain (%)
Gr on ODTS	1.78 ~ 5.58	~0 to 0.21
Gr on FOTS	8.87 ~ 12.88	-0.18 to 0.05
Gr on MAL	2.28 ~ 10.19	-0.14 ~ 0.11

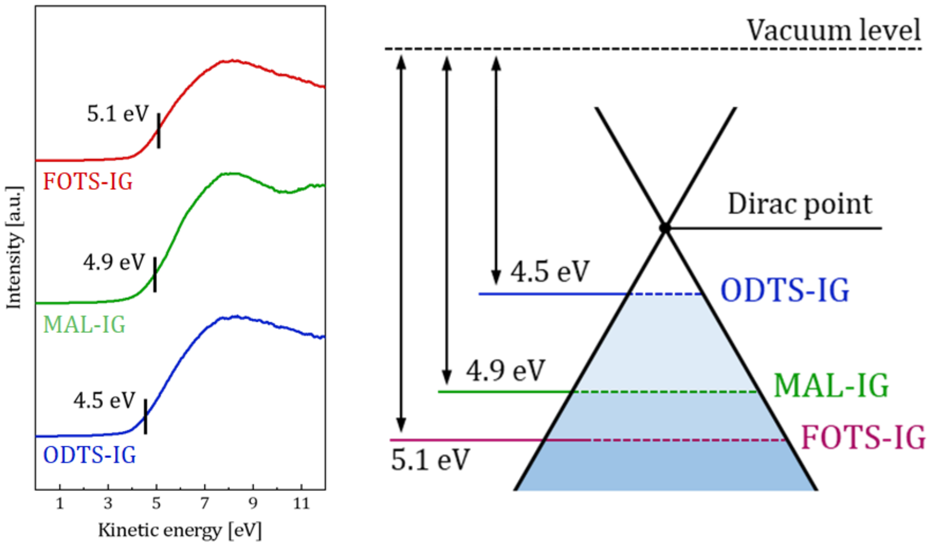

Figure S2. UPS spectra at the low kinetic energy region for ODTS-IG, MAL-IG, and FOTS-IG, respectively, and the corresponding measured Fermi level of ODTS-IG, MAL-IG, and FOTS-IG.

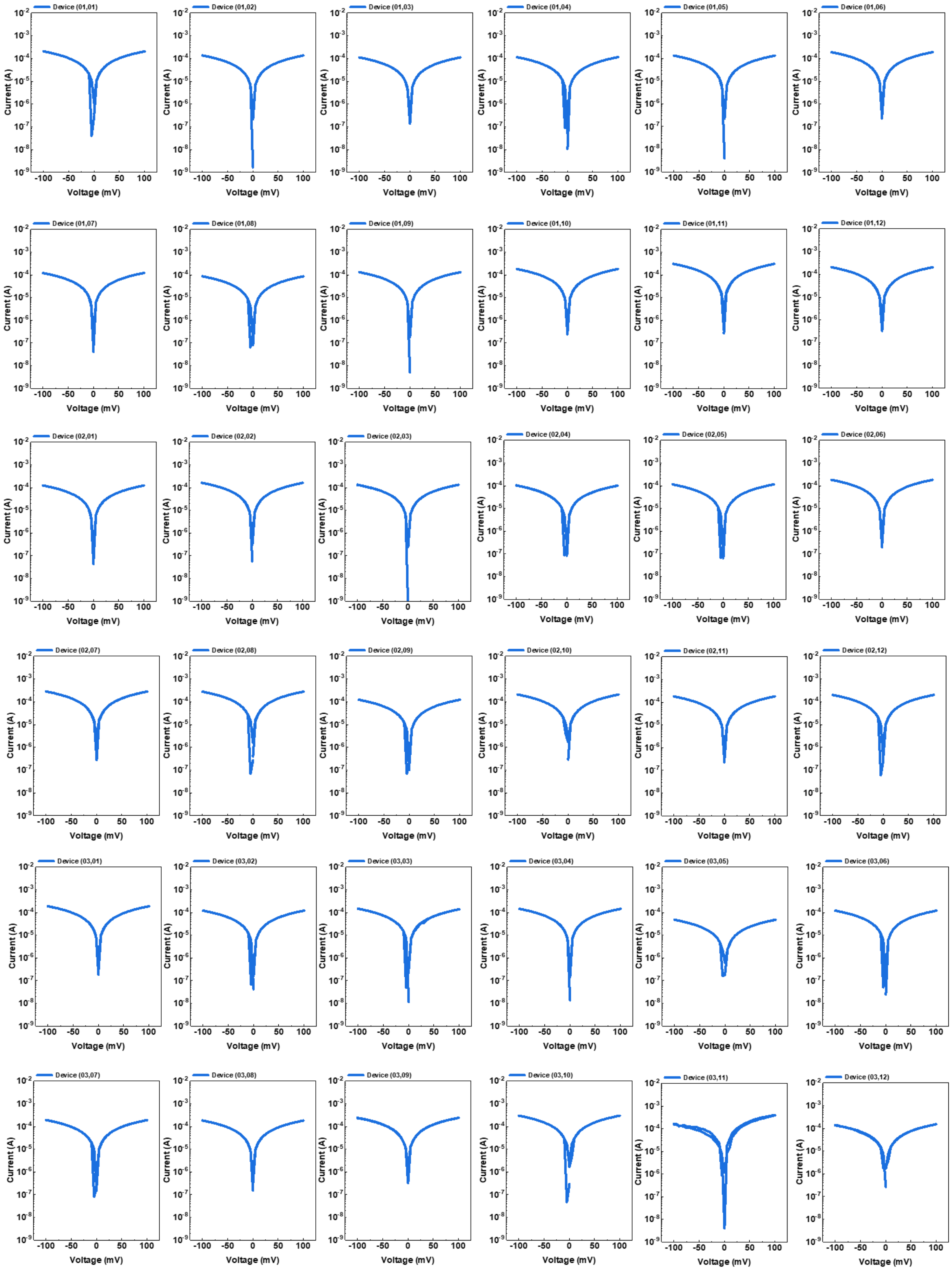
Device (01,01)
Device (01,02)
Device (01,03)
Device (01,04)
Device (01,05)
Device (01,06)
Device (01,07)
Device (01,08)
Device (01,09)
Device (01,10)
Device (01,11)
Device (01,12)
Device (02,01)
Device (02,02)
Device (02,03)
Device (02,04)
Device (02,05)
Device (02,06)
Device (02,07)
Device (02,08)
Device (02,09)
Device (02,10)
Device (02,11)
Device (02,12)
Device (03,01)
Device (03,02)
Device (03,03)
Device (03,04)
Device (03,05)
Device (03,06)
Device (03,07)
Device (03,08)
Device (03,09)
Device (03,10)
Device (03,11)
Device (03,12)
Current (A)
Voltage (mV)

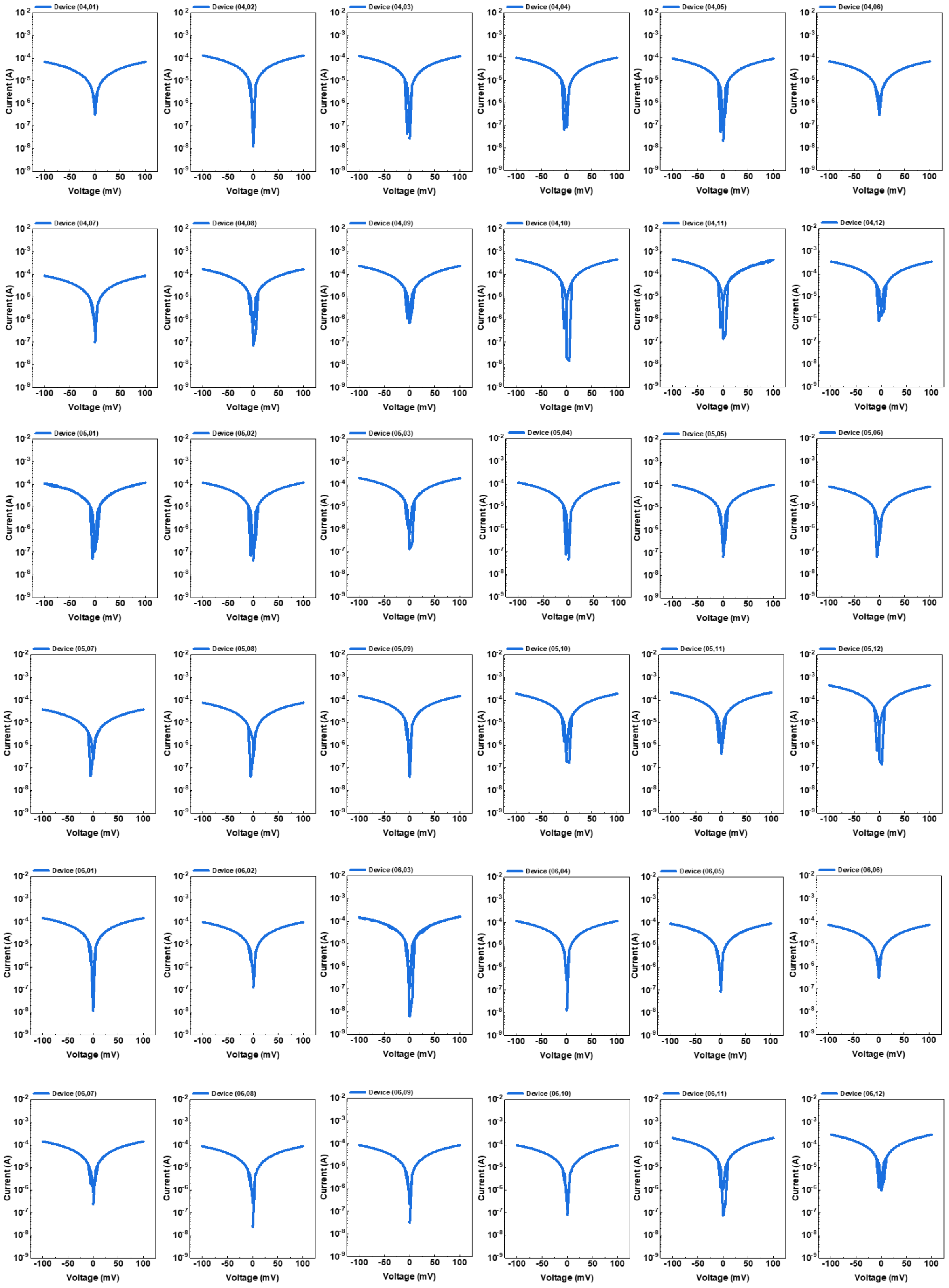

Device (04,01)
Device (04,02)
Device (04,03)
Device (04,04)
Device (04,05)
Device (04,06)
Device (04,07)
Device (04,08)
Device (04,09)
Device (04,10)
Device (04,11)
Device (04,12)
Device (05,01)
Device (05,02)
Device (05,03)
Device (05,04)
Device (05,05)
Device (05,06)
Device (05,07)
Device (05,08)
Device (05,09)
Device (05,10)
Device (05,11)
Device (05,12)
Device (06,01)
Device (06,02)
Device (06,03)
Device (06,04)
Device (06,05)
Device (06,06)
Device (06,07)
Device (06,08)
Device (06,09)
Device (06,10)
Device (06,11)
Device (06,12)
Current (A)
Voltage (mV)

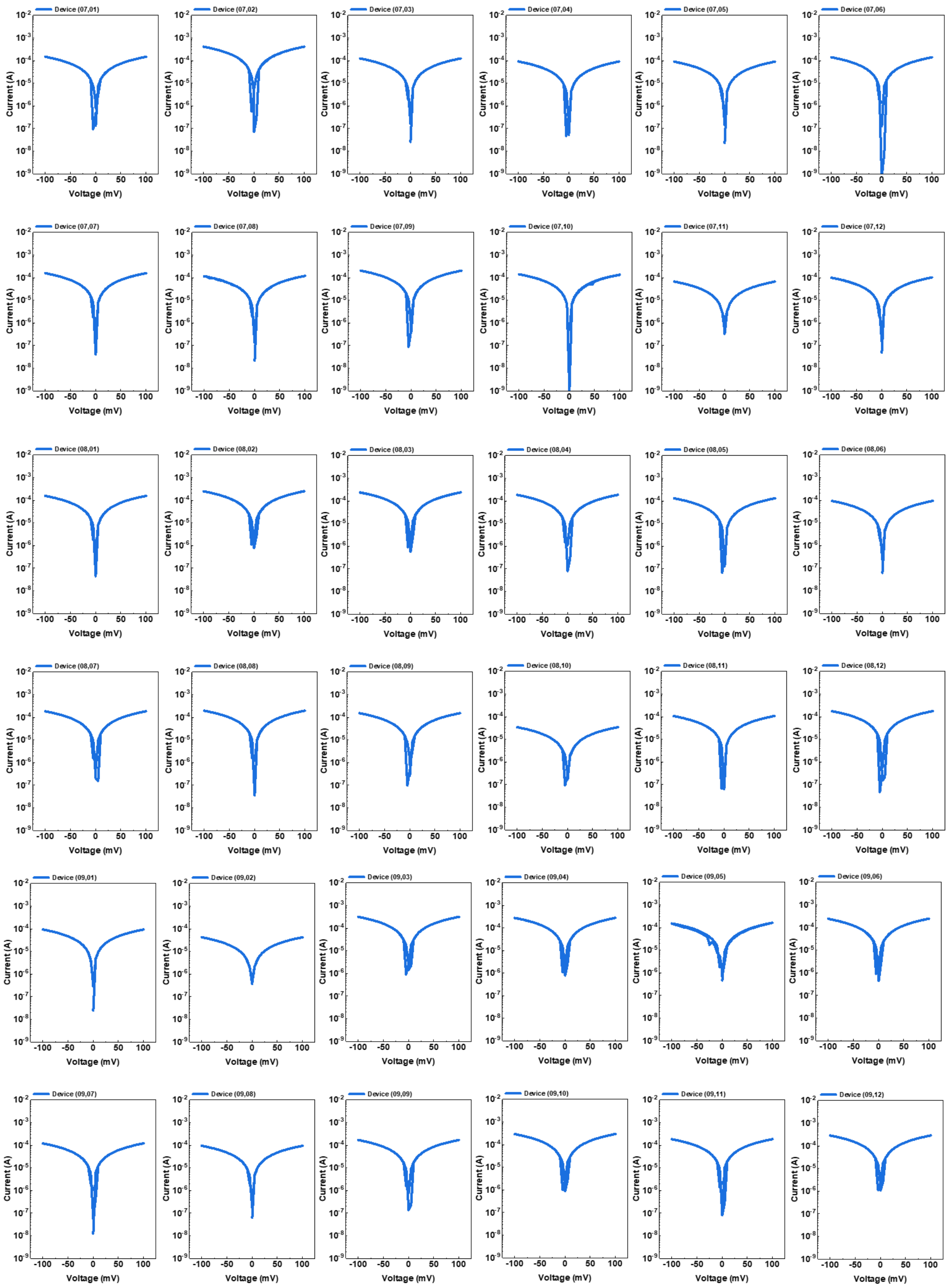
Device (07,01)
Device (07,02)
Device (07,03)
Device (07,04)
Device (07,05)
Device (07,06)
Device (07,07)
Device (07,08)
Device (07,09)
Device (07,10)
Device (07,11)
Device (07,12)
Device (08,01)
Device (08,02)
Device (08,03)
Device (08,04)
Device (08,05)
Device (08,06)
Device (08,07)
Device (08,08)
Device (08,09)
Device (08,10)
Device (08,11)
Device (08,12)
Device (09,01)
Device (09,02)
Device (09,03)
Device (09,04)
Device (09,05)
Device (09,06)
Device (09,07)
Device (09,08)
Device (09,09)
Device (09,10)
Device (09,11)
Device (09,12)
Current (A)
Voltage (mV)

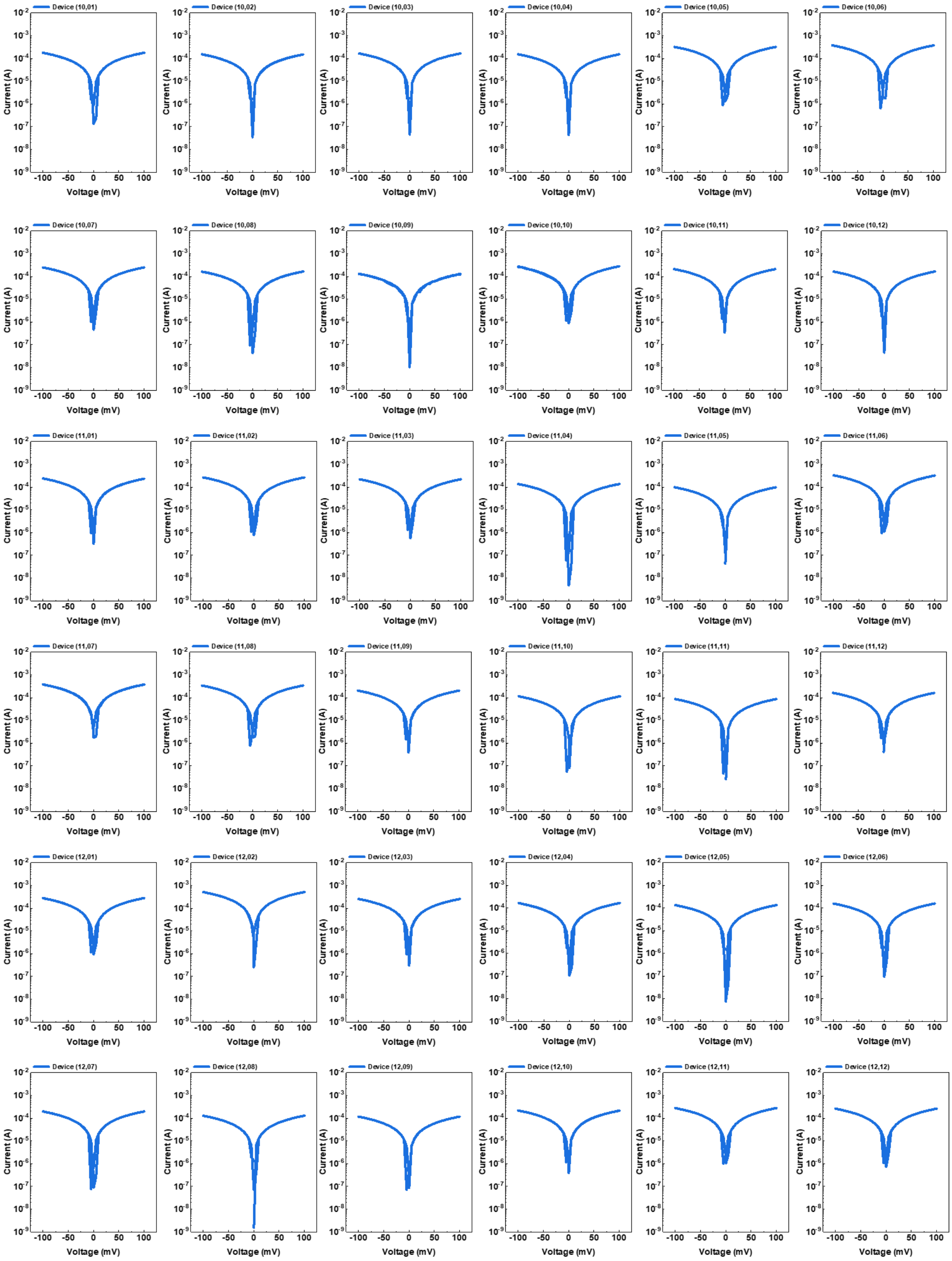

Figure S3. 144 I–V characteristic graphs of ODTS-IG at a 12 × 12 array.

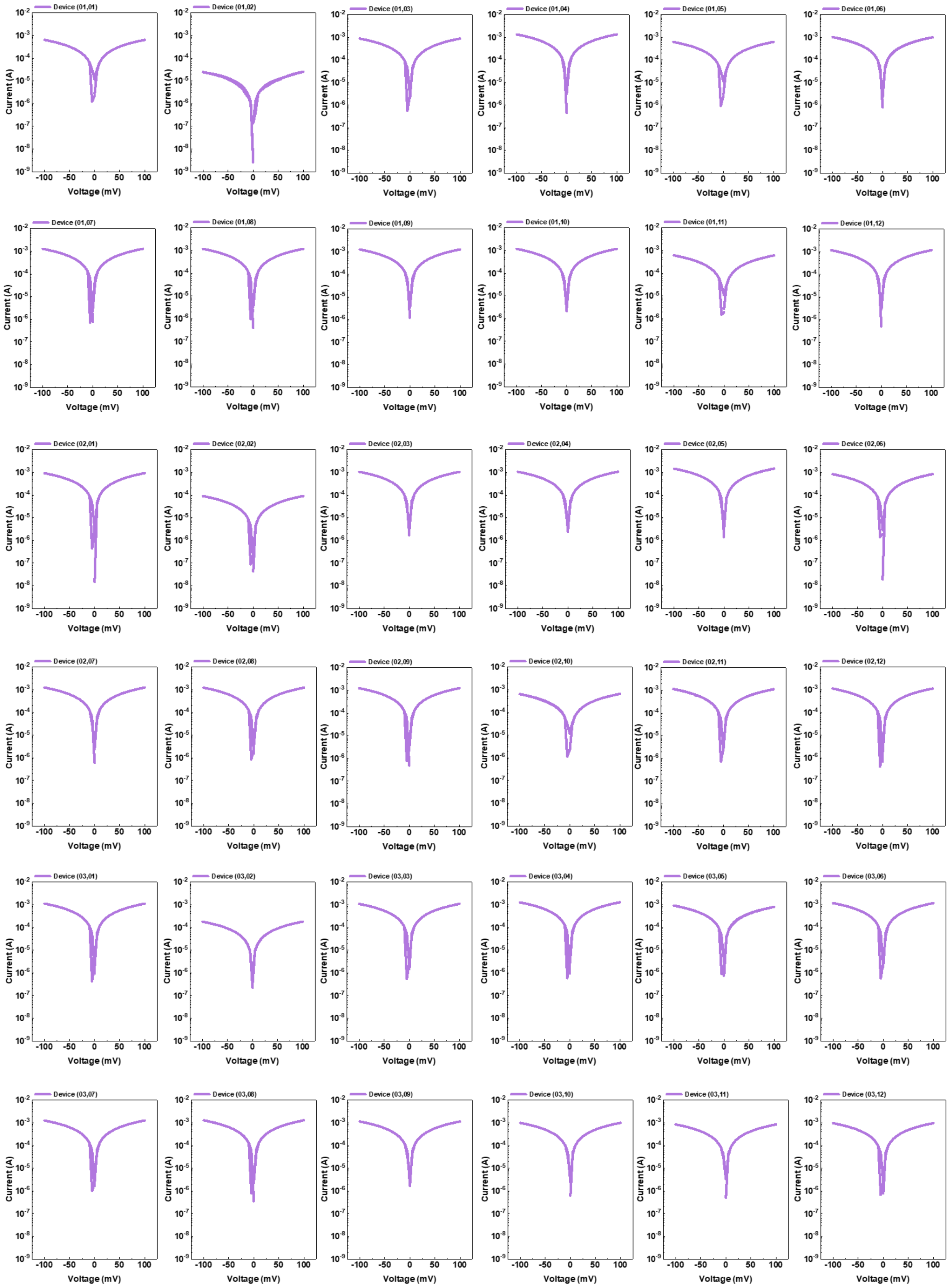
Device (01,01)
Device (01,02)
Device (01,03)
Device (01,04)
Device (01,05)
Device (01,06)
Device (01,07)
Device (01,08)
Device (01,09)
Device (01,10)
Device (01,11)
Device (01,12)
Device (02,01)
Device (02,02)
Device (02,03)
Device (02,04)
Device (02,05)
Device (02,06)
Device (02,07)
Device (02,08)
Device (02,09)
Device (02,10)
Device (02,11)
Device (02,12)
Device (03,01)
Device (03,02)
Device (03,03)
Device (03,04)
Device (03,05)
Device (03,06)
Device (03,07)
Device (03,08)
Device (03,09)
Device (03,10)
Device (03,11)
Device (03,12)
Current (A)
Voltage (mV)

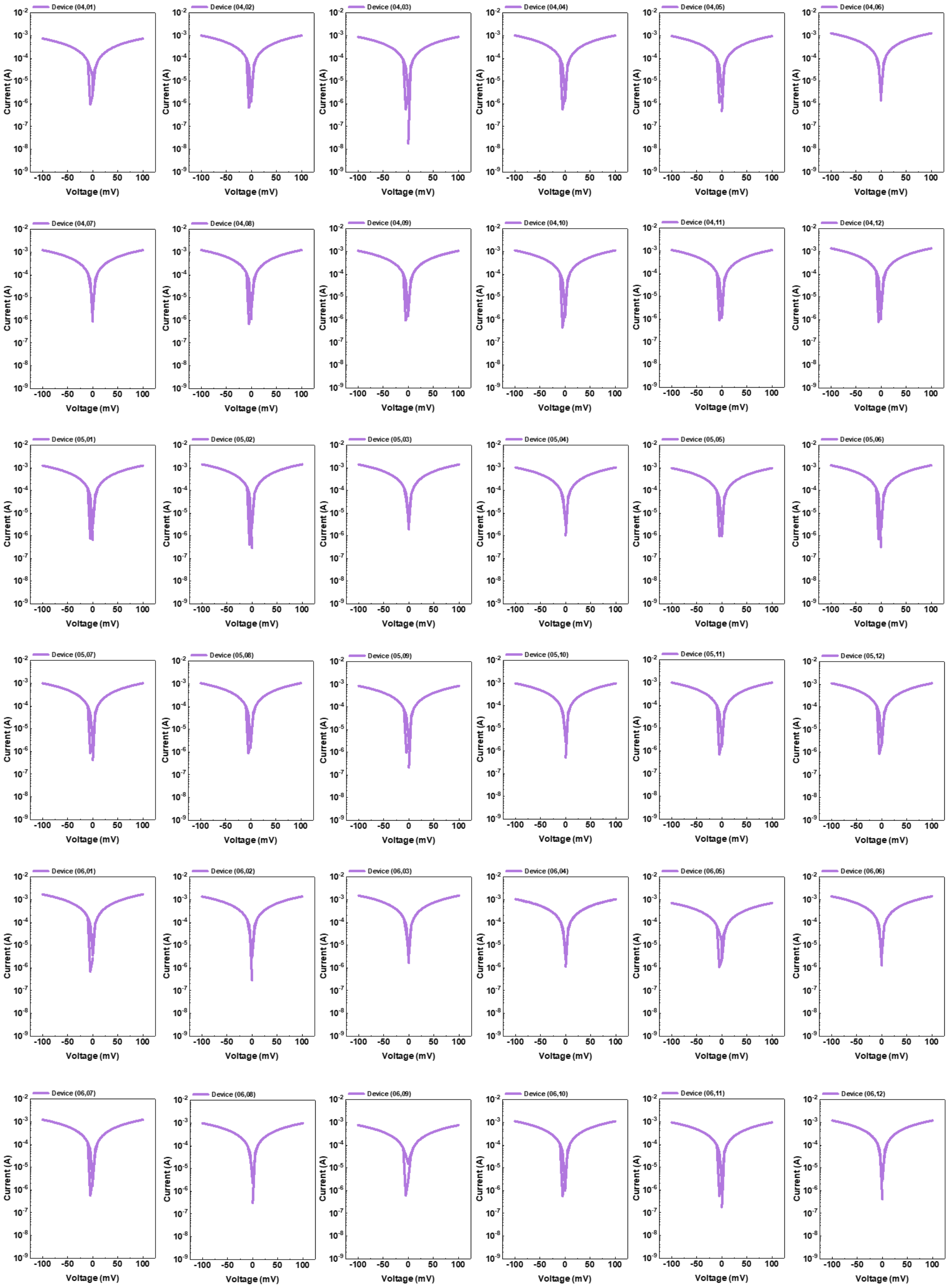
Device (04,01)
Device (04,02)
Device (04,03)
Device (04,04)
Device (04,05)
Device (04,06)
Device (04,07)
Device (04,08)
Device (04,09)
Device (04,10)
Device (04,11)
Device (04,12)
Device (05,01)
Device (05,02)
Device (05,03)
Device (05,04)
Device (05,05)
Device (05,06)
Device (05,07)
Device (05,08)
Device (05,09)
Device (05,10)
Device (05,11)
Device (05,12)
Device (06,01)
Device (06,02)
Device (06,03)
Device (06,04)
Device (06,05)
Device (06,06)
Device (06,07)
Device (06,08)
Device (06,09)
Device (06,10)
Device (06,11)
Device (06,12)
Current (A)
Voltage (mV)

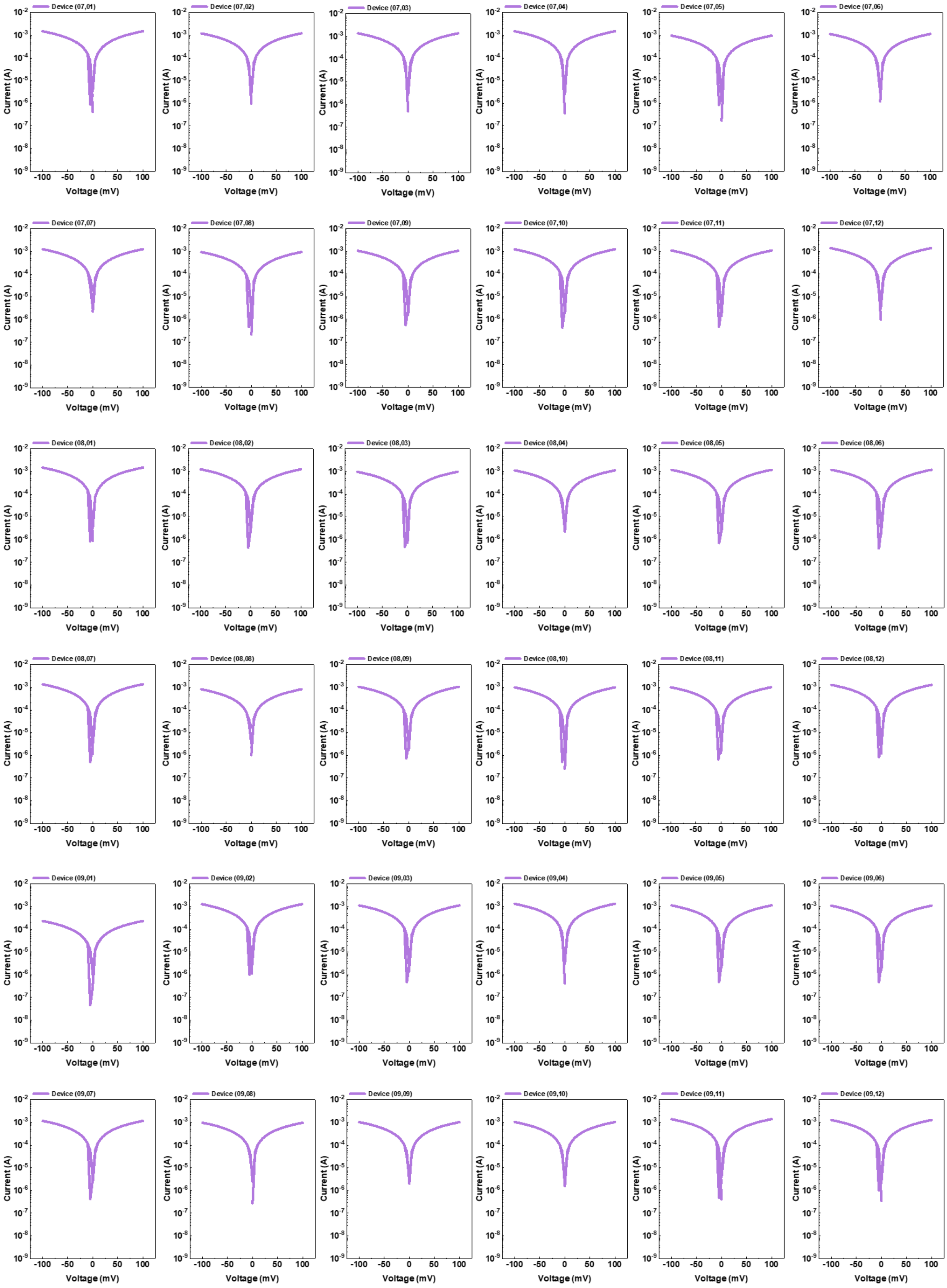
Device (07,01)
Device (07,02)
Device (07,03)
Device (07,04)
Device (07,05)
Device (07,06)
Device (07,07)
Device (07,08)
Device (07,09)
Device (07,10)
Device (07,11)
Device (07,12)
Device (08,01)
Device (08,02)
Device (08,03)
Device (08,04)
Device (08,05)
Device (08,06)
Device (08,07)
Device (08,08)
Device (08,09)
Device (08,10)
Device (08,11)
Device (08,12)
Device (09,01)
Device (09,02)
Device (09,03)
Device (09,04)
Device (09,05)
Device (09,06)
Device (09,07)
Device (09,08)
Device (09,09)
Device (09,10)
Device (09,11)
Device (09,12)
Current (A)
Voltage (mV)

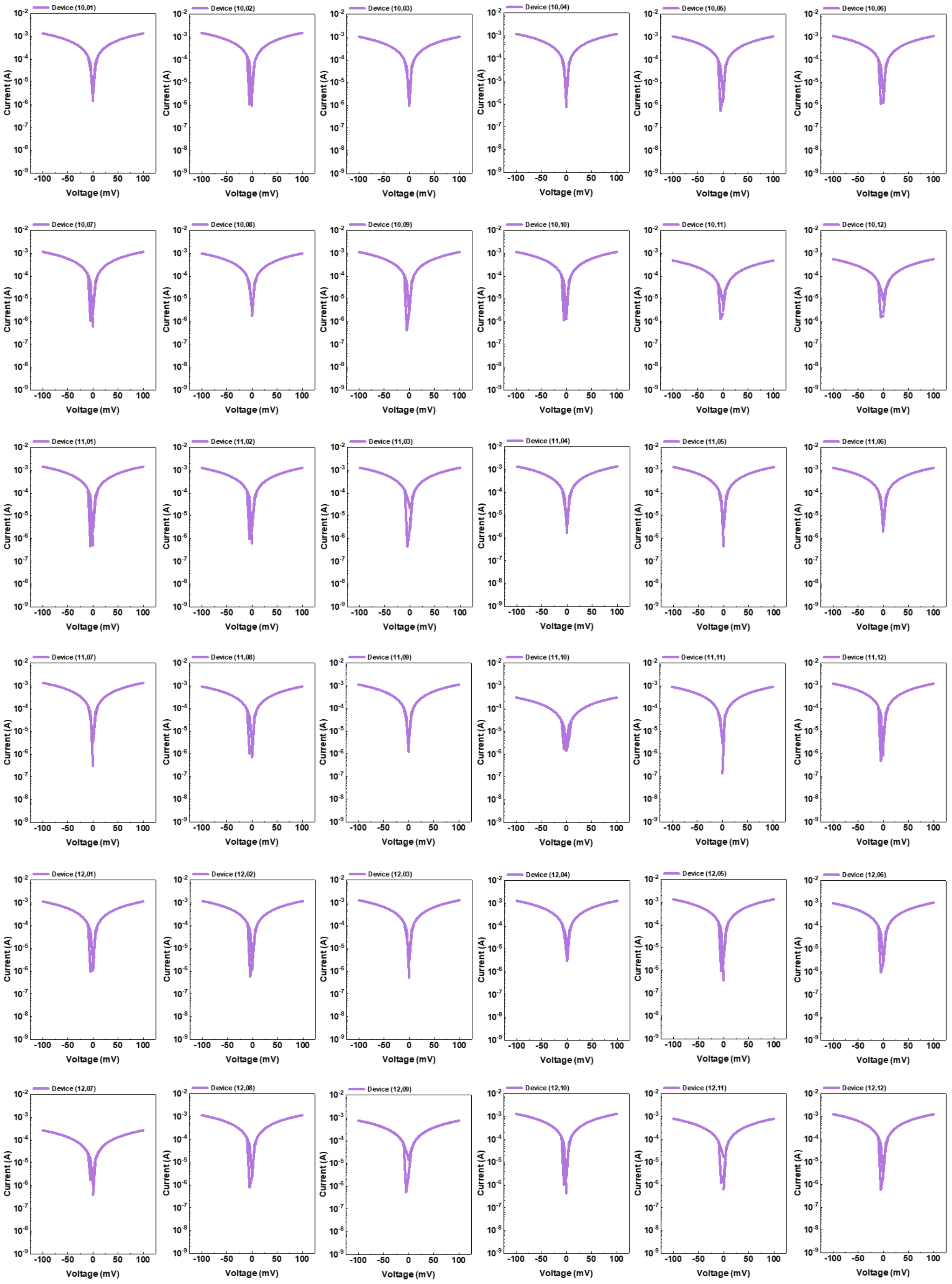

Figure S4. 144 I–V characteristic graphs of MAL-IG at a 12 × 12 array.

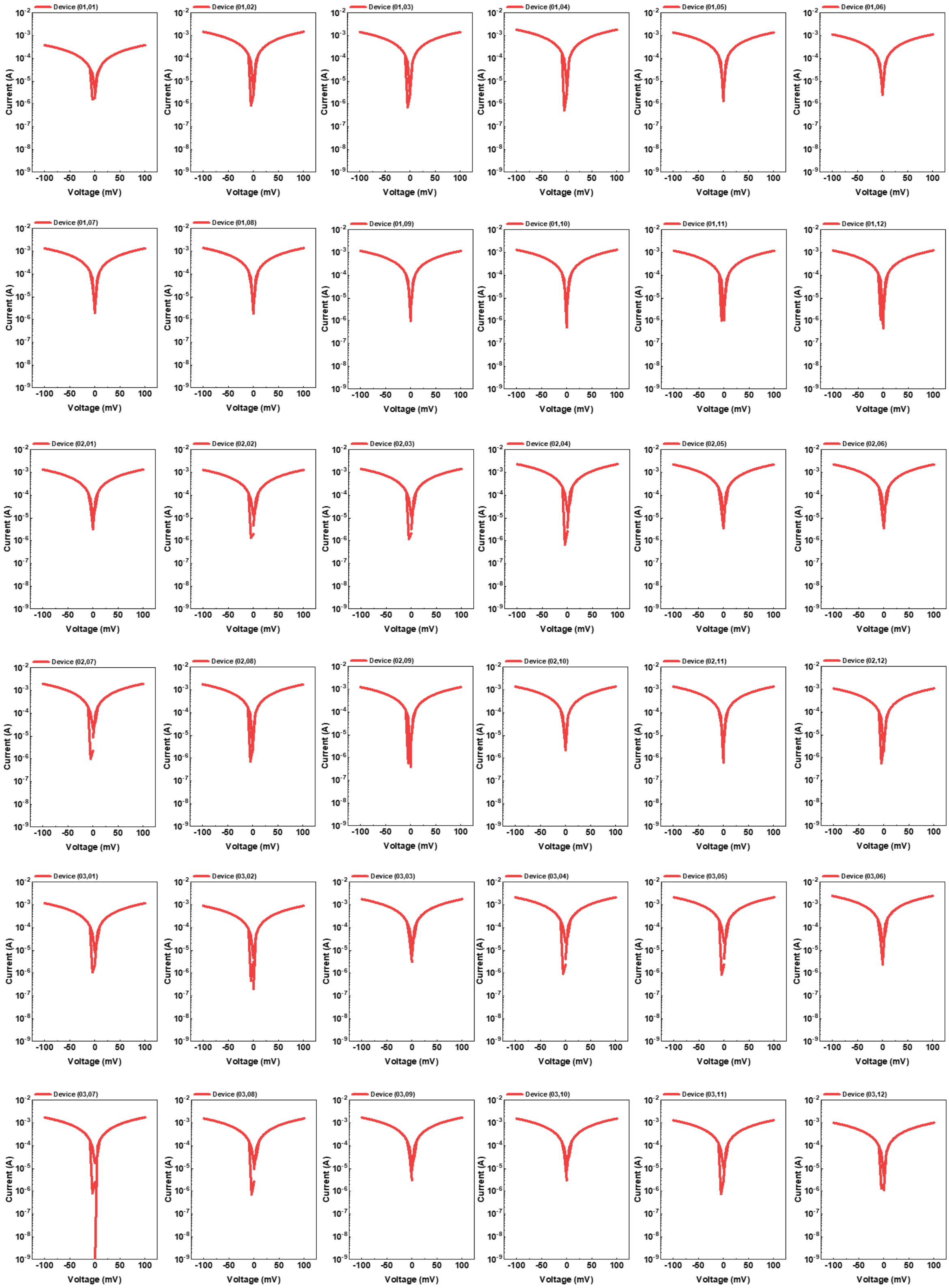
Device (01,01)
Device (01,02)
Device (01,03)
Device (01,04)
Device (01,05)
Device (01,06)
Device (01,07)
Device (01,08)
Device (01,09)
Device (01,10)
Device (01,11)
Device (01,12)
Device (02,01)
Device (02,02)
Device (02,03)
Device (02,04)
Device (02,05)
Device (02,06)
Device (02,07)
Device (02,08)
Device (02,09)
Device (02,10)
Device (02,11)
Device (02,12)
Device (03,01)
Device (03,02)
Device (03,03)
Device (03,04)
Device (03,05)
Device (03,06)
Device (03,07)
Device (03,08)
Device (03,09)
Device (03,10)
Device (03,11)
Device (03,12)
Current (A)
10^{-2}
10^{-3}
10^{-4}
10^{-5}
10^{-6}
10^{-7}
10^{-8}
10^{-9}
Voltage (mV)
-100
-50
0
50
100

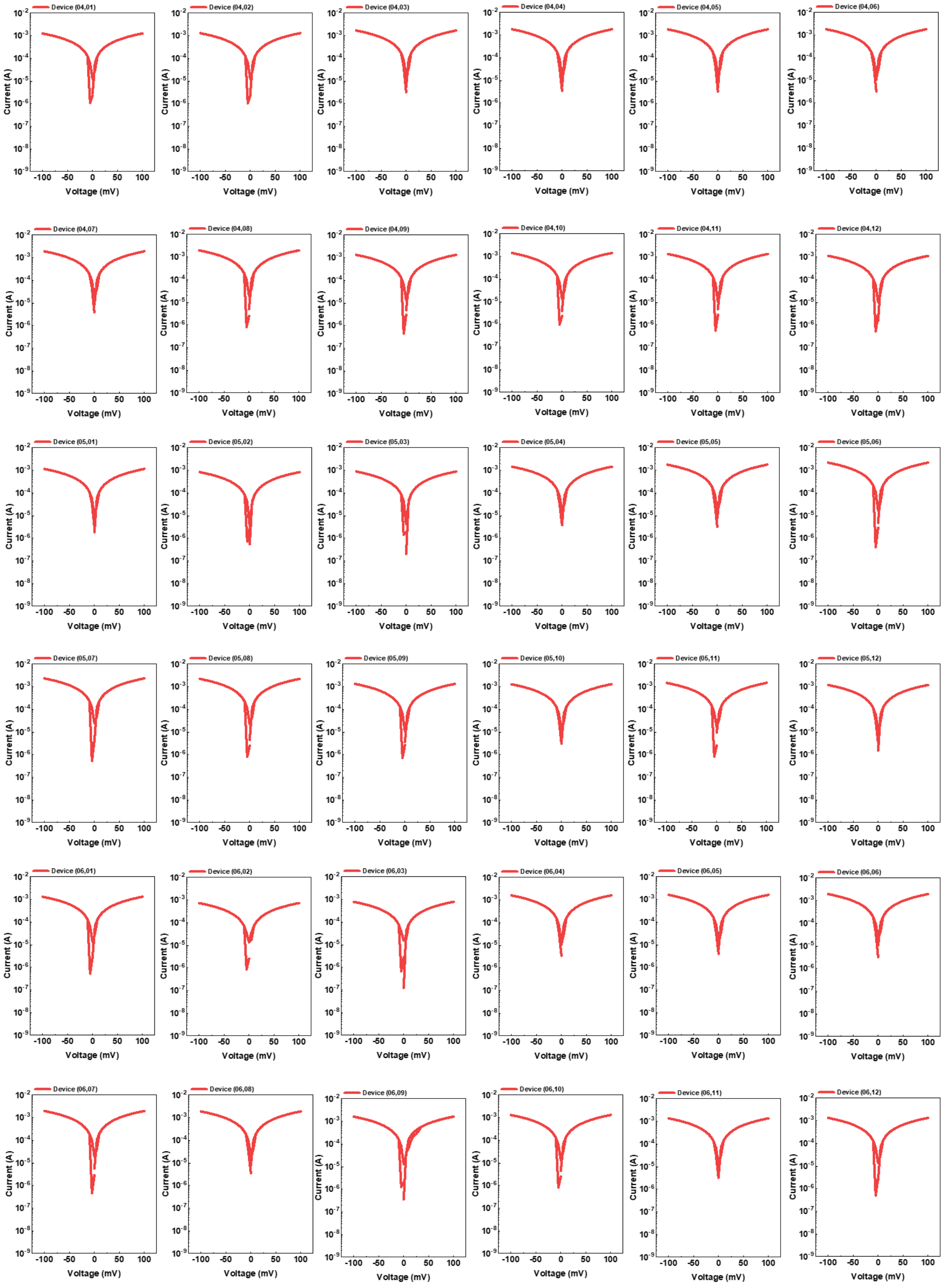
Device (04,01)
Device (04,02)
Device (04,03)
Device (04,04)
Device (04,05)
Device (04,06)
Device (04,07)
Device (04,08)
Device (04,09)
Device (04,10)
Device (04,11)
Device (04,12)
Device (05,01)
Device (05,02)
Device (05,03)
Device (05,04)
Device (05,05)
Device (05,06)
Device (05,07)
Device (05,08)
Device (05,09)
Device (05,10)
Device (05,11)
Device (05,12)
Device (06,01)
Device (06,02)
Device (06,03)
Device (06,04)
Device (06,05)
Device (06,06)
Device (06,07)
Device (06,08)
Device (06,09)
Device (06,10)
Device (06,11)
Device (06,12)
Current (A)
Voltage (mV)

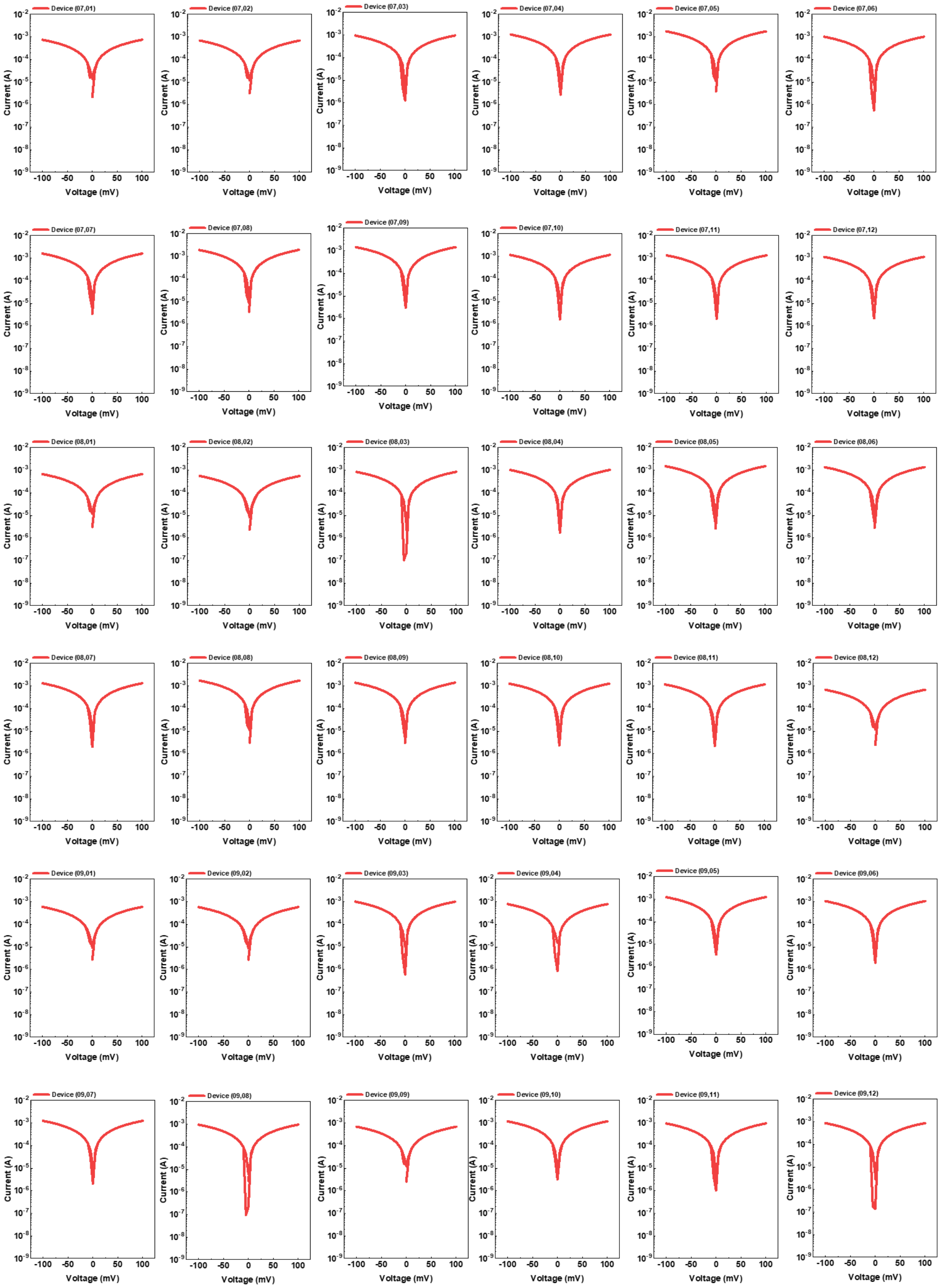
Device (07,01)
Device (07,02)
Device (07,03)
Device (07,04)
Device (07,05)
Device (07,06)
Device (07,07)
Device (07,08)
Device (07,09)
Device (07,10)
Device (07,11)
Device (07,12)
Device (08,01)
Device (08,02)
Device (08,03)
Device (08,04)
Device (08,05)
Device (08,06)
Device (08,07)
Device (08,08)
Device (08,09)
Device (08,10)
Device (08,11)
Device (08,12)
Device (09,01)
Device (09,02)
Device (09,03)
Device (09,04)
Device (09,05)
Device (09,06)
Device (09,07)
Device (09,08)
Device (09,09)
Device (09,10)
Device (09,11)
Device (09,12)
Current (A)
Voltage (mV)

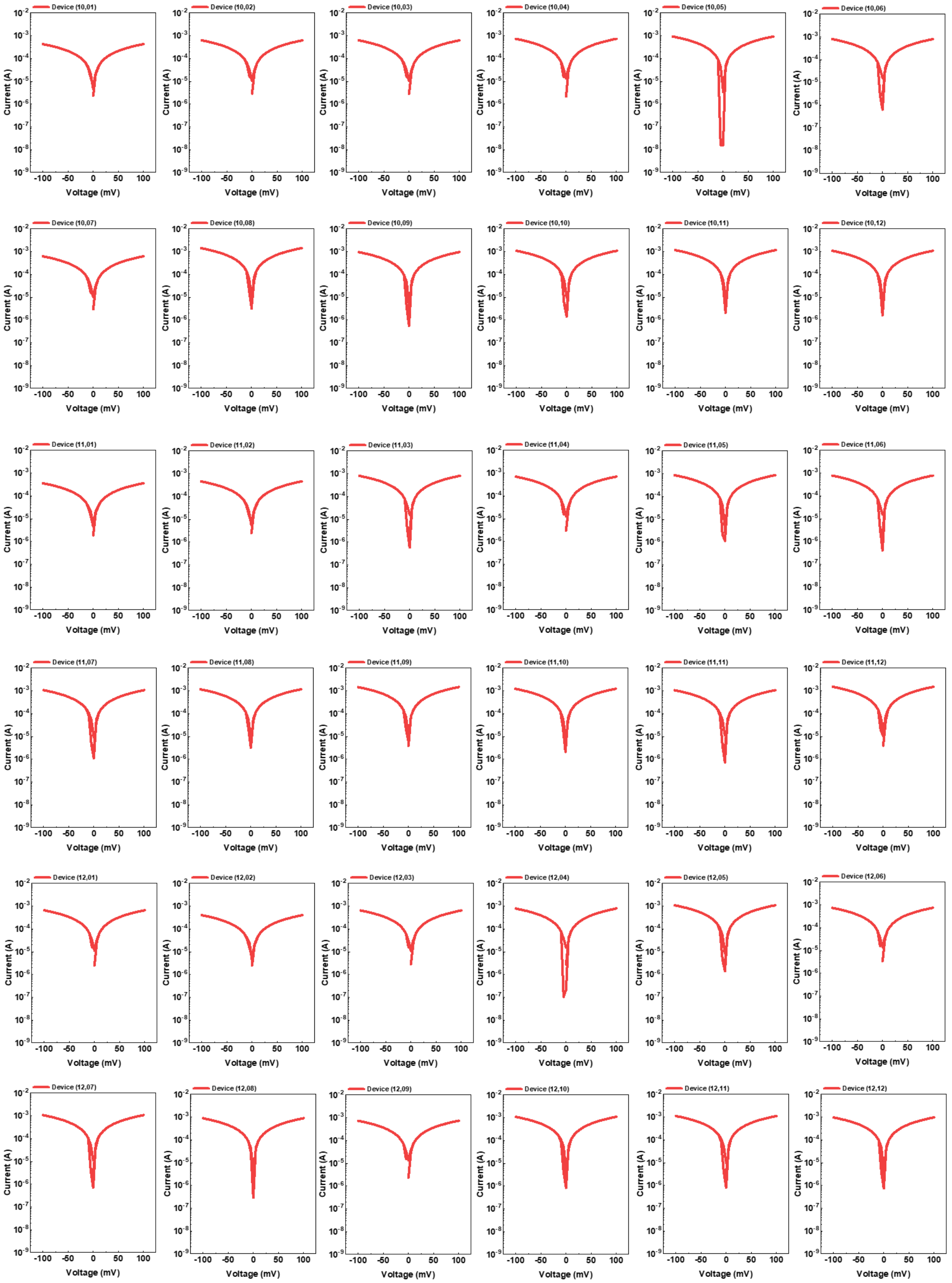

Figure S5. 144 I–V characteristic graphs of FOTS-IG at a 12 × 12 array.

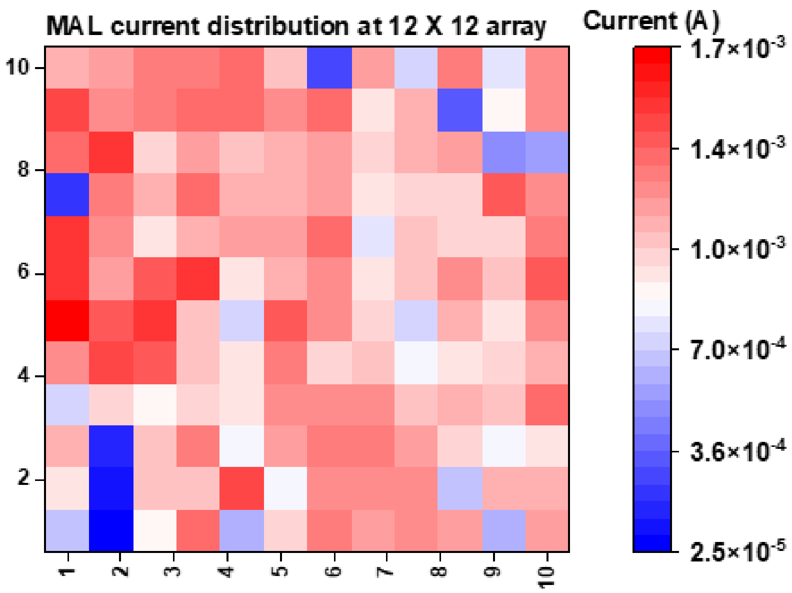

Figure S6. Mapped current value when 100 mV voltage is applied in a 12 × 12 array with MAL-IG.

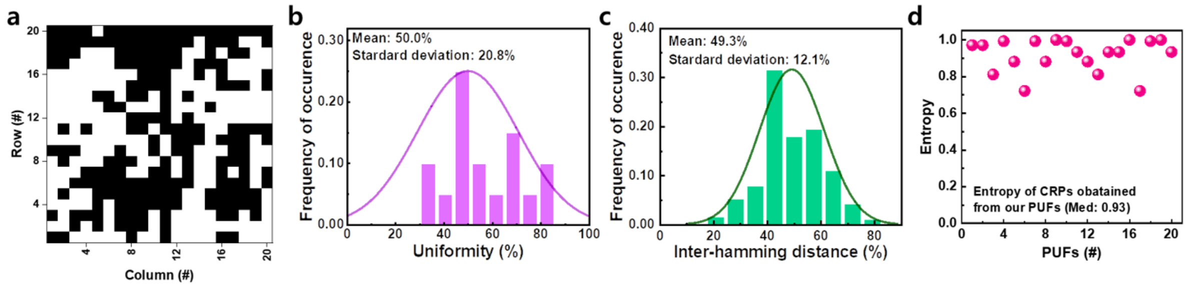

Figure S7. a) Security key at 20 × 20 array. b) The uniformity for randomness and c) the inter-HD for uniqueness at batch-to-batch. d) The entropy of challenge-response pairs (CRPs) obtained from the 20 × 20 array PUF.

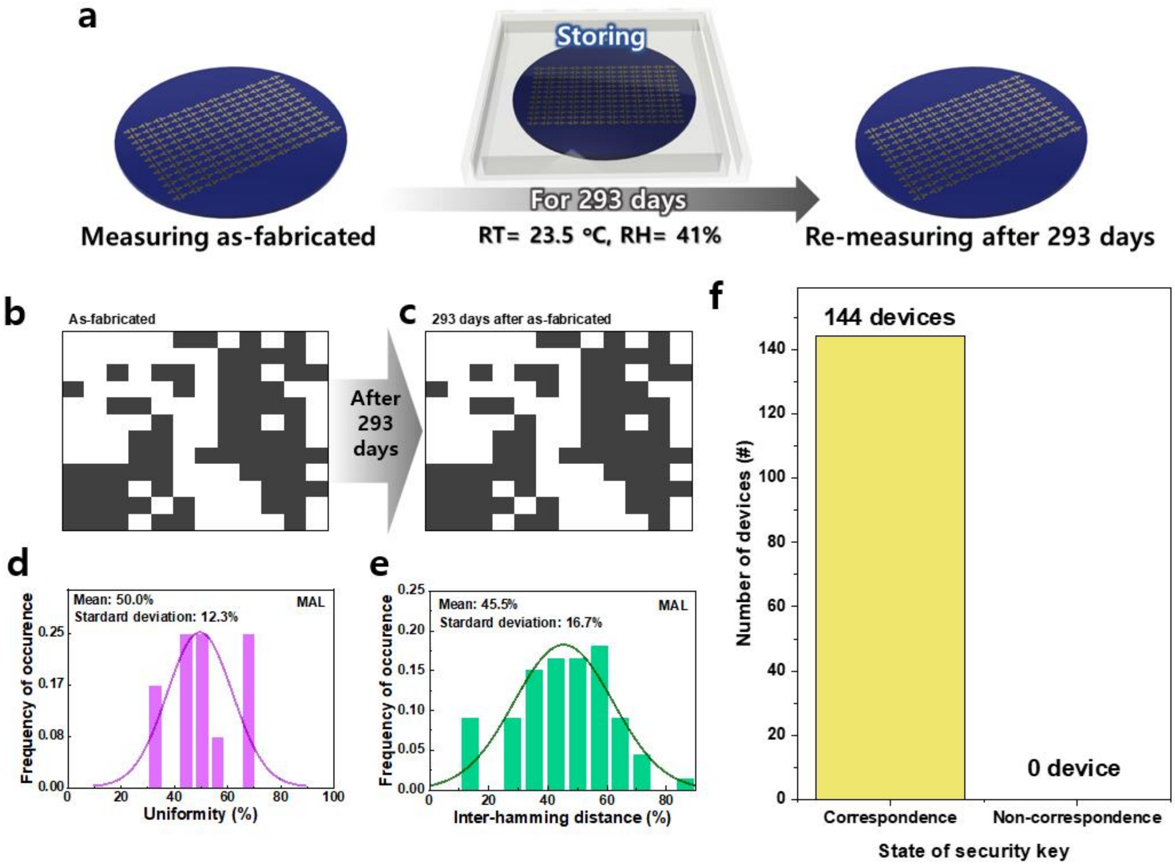

Figure S8. a) Device storage environment and period. Security key for b) as-fabricated and c) 293 days after as-fabricated. Evaluation of key for d) uniformity and e) inter-HD. f) Degree of coincidence for security key between as-fabricated and 293 days after.

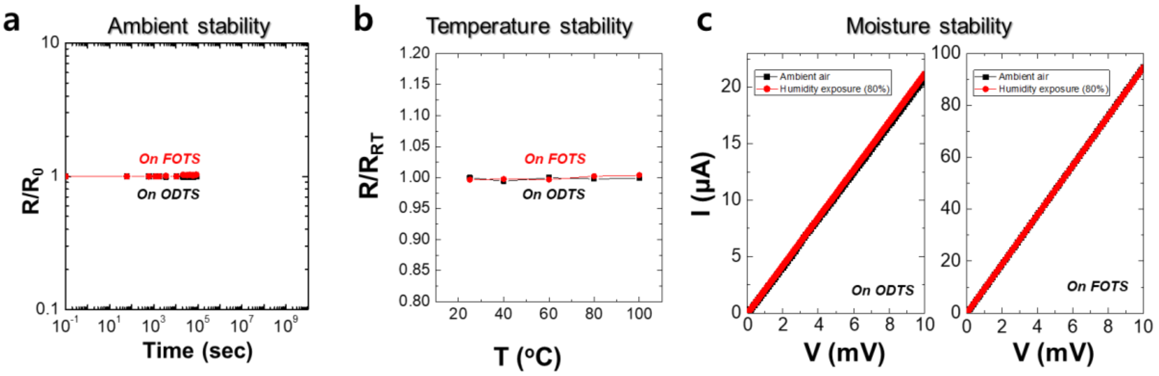

Figure S9. a) Ambient, b) temperature, and c) moisture stability of graphene transferred onto ODTS- and FOTS-treated SiO_2/Si substrate.

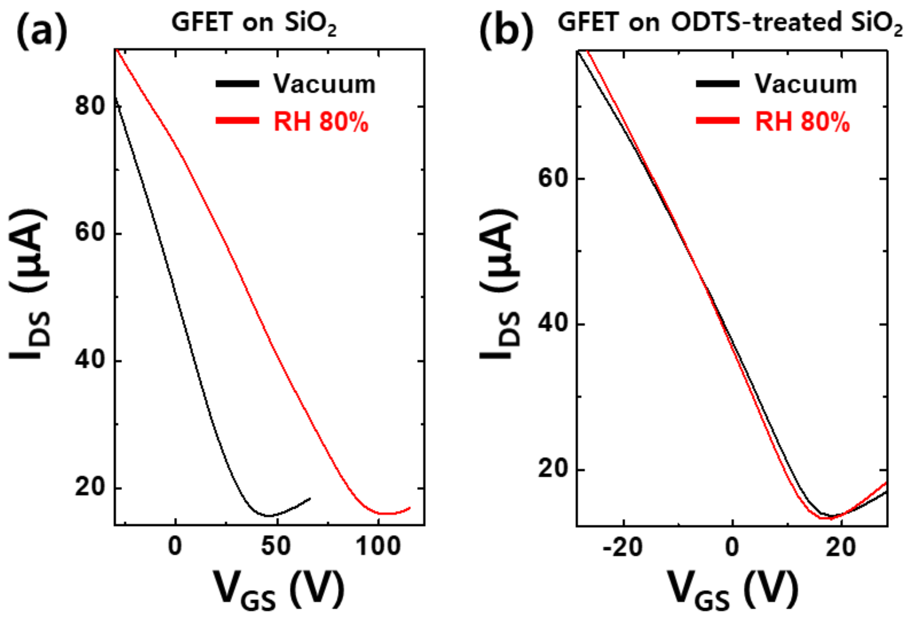

Figure S10. The gate-voltage-dependent drain current measurement (V_{DS}= 10 mV) of graphene FETs on a) bare SiO_2/Si substrate and b) ODTS-treated SiO_2/Si substrate. The black and red lines indicate the curves measured under vacuum conditions and at a relative humidity (RH) of approximately 80%, respectively.

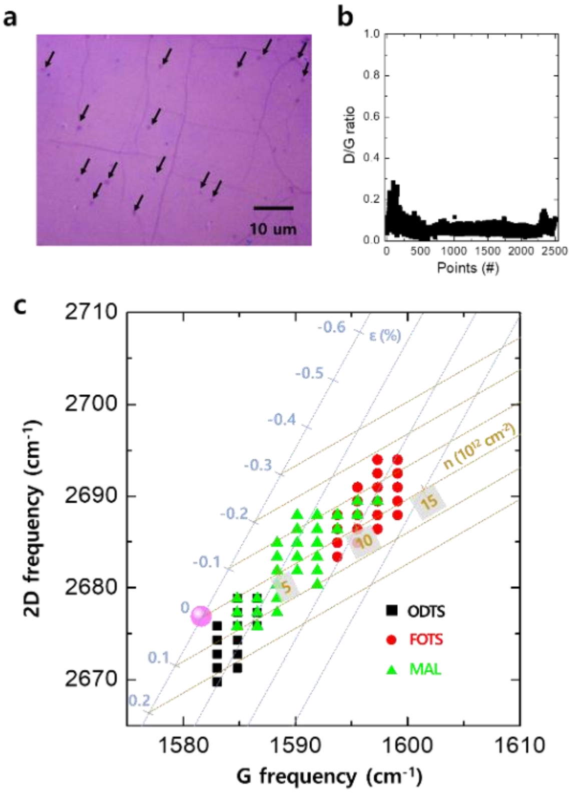

Figure S11. a) Optical microscope image and b) D/G ratio of CVD-grown graphene transferred onto SiO_2/Si substrate. c) Correlation between the frequencies of the G-band (ω_G) and 2D-band (ω_{2D}) of graphene, extracted from Raman spectra. To investigate the defects in the CVD-grown graphene transferred onto a SiO_2/Si substrate, Raman spectroscopy was performed in **Figure S11**. In Figure S11b, we present the D/G ratio obtained from the 2,500 Raman spectra (within the area of 10,000 μm^2). The D and G peaks correspond to the sp^3 dangling bond and the in-plane vibrations of sp^2 bonded carbon in graphene, respectively. Therefore, the D/G ratio serves as an indicator of the sp^3/sp^2 carbon ratio. The results indicate that the average D/G ratio is calculated to be 0.063 with a standard deviation of 0.032.

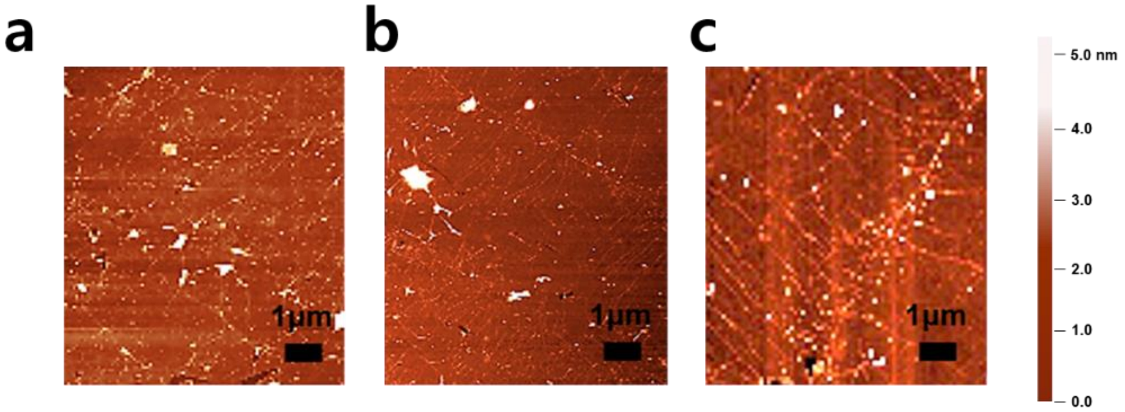

Figure S12. AFM images of graphene transferred onto a) ODTS-, b) FOTS-, and c) MAL-treated SiO_2/Si substrates. To examine the presence of folded graphene in our samples, we performed AFM measurements on three distinct graphene films, as shown in **Figure S12**. Our observations indicate a minimal occurrence of folded graphene in these samples. As a result, we can confidently state that the influence of wrinkles on the electrical properties in our system is insignificant. It is important to note, however, that AFM analysis is limited to a local area, and thus the possibility of folded graphene cannot be entirely ruled out. Nevertheless, folded graphene is considered acceptable as it can have a positive impact by amplifying the variation in resistance values.

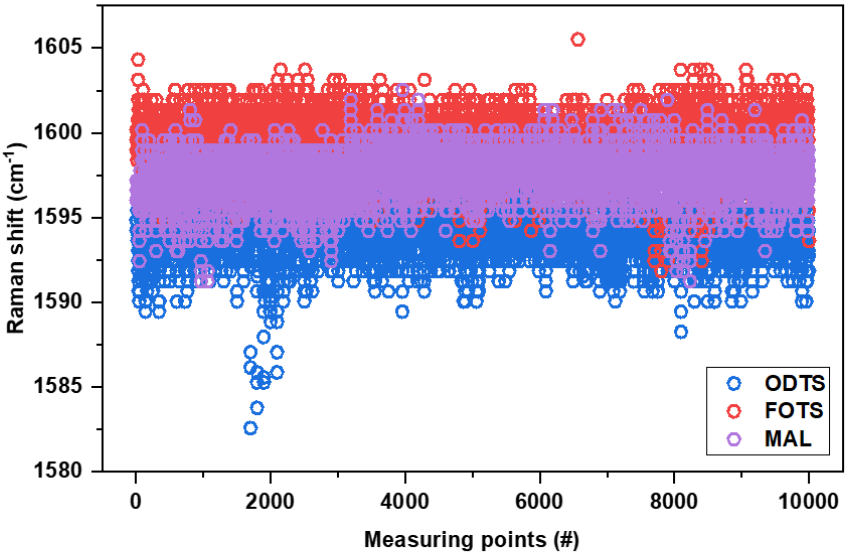

Figure S13. G band Raman spectroscopy of 10,000 points measured on ODTS, FOTS, and MAL-embedded graphene

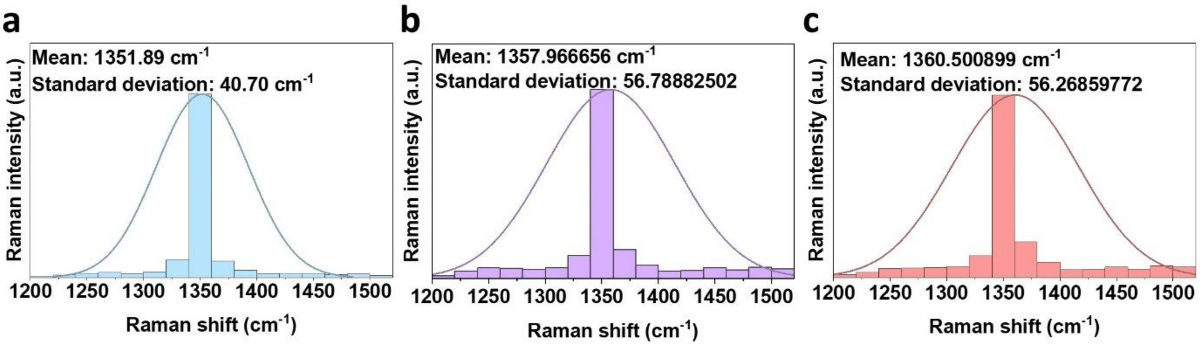

Figure S14. Raman spectroscopy normal distribution graph at graphene G band by inserting SAM layer of a) ODTS, b) MAL, c) FOTS.

Table S2. Attack prediction rate at each doping state of the device evaluated by ML training.

**Devices**	**ODTS-inserted graphene**	**MAL-inserted graphene**	**FOTS-inserted graphene**
Predict rate (%)	100	10.33	44.81

Table S3. Mechanism and evaluation of PUFs and the device performance according to PUFs type using graphene.

PUF type	Material	Mechanism	Operation voltage	Evaluation parameter	Prediction accuracy	Data number	Security mode	Long-term stability	Ref
Strain PUF	Graphene	Stochastic strain-response on piezoelectric lead zirconate titanate (PZT)	42 V	Entropy, Hamming distance, Correlation Coefficient	40%	400	Single	5 days	[S1]
Resistive PUF	Graphene	The “imperfect” van der Waals interface of CVD-synthesized graphene	1 V	Hamming weight, inter-Hamming distance, entropy	65%	576	Single	N/A	[S2]
Image PUF	Graphene	Interplay between LSPR and Fabry−Pérot modes to enrich response signatures forming the random colored patterns, reflection spectra, and Raman spectra	0 V	Inter-Hamming distance, intra-hamming distance	N/A	N/A	Quad	N/A	[S3]
Image PUF	Graphene	In situ crystallization of chaotic phosphorescent patterns	0 V	Mean, standard deviation	N/A	480	Single	N/A	[S4]
Resistive PUF	Graphene	Impurity-dominated diffusive transport that introduces natural randomness	5 V	Uniqueness, Correlation	52.5%	240	Single	7 hours	[S5]
Resistive PUF	Graphene	Two self-assembled monolayer materials with opposite dipole inserted at the graphene to dope the graphene irregularly	0 V, 100 mV	Uniqueness, inter-Hamming distance, entropy, reconfiguration	10.33%	10,000, 144	Dual	293 days	This work

Supporting reference

[S1] S. Ghosh, Y. Zheng, S. S. Radhakrishnan, T. F. Schranghamer, S. Das, Nano Lett. 2023.

[S2] B. Liu, J. Ma, H. H. Tai, D. Verma, M. Sahoo, Y.-F. Chang, H. Liang, S. Feng, L.-J. Li, T.-H. Hou, ACS Appl. Electron. Mater. 2023, 5, 714.

[S3] S. Jiang, S.-H. Kim, C.-S. Park, W.-B. Lee, S.-S. Lee, ACS Appl. Mater. Interfaces 2022, 14, 39240.

[S4] H. Im, J. Yoon, J. Choi, J. Kim, S. Baek, D. H. Park, W. Park, S. Kim, Adv. Mater. 2021, 33, 2102542.

[S5] A. Dodda, S. Subbulakshmi Radhakrishnan, T. F. Schranghamer, D. Buzzell, P. Sengupta, S. Das, Nat. Electron. 2021, 4, 364.
